# The association between socioeconomic position and depression or suicidal ideation in low- and middle-income countries in Southeast Asia: a systematic review and meta-analysis

**DOI:** 10.1186/s12889-024-20986-9

**Published:** 2024-12-18

**Authors:** Lucy Barrass, Elisha Joshi, Joshua Dawe, Bruna Rubbo, Maria Theresa Redaniel, Lucy Riglin, Nanette R. Lee, Laura D. Howe, Duleeka Knipe

**Affiliations:** 1 Population Health Sciences, Bristol Medical School, University of Bristol, Bristol, UK; 2 NIHR ARC West, Population Health Sciences, University of Bristol, Bristol, UK; 3 The Wolfson Centre for Young People’s Mental Health and Division of Psychological Medicine and Clinical Neurosciences, MRC Centre for Neuropsychiatric Genetics and Genomics, Cardiff University, Cardiff, UK; 4 Office of Population Studies Foundation, University of San Carlos, Cebu City, Philippines; 5 South Asian Clinical Toxicology Research Collaboration, Faculty of Medicine, University of Peradeniya, Peradeniya, Sri Lanka

**Keywords:** Review, Depression, Suicidal ideation, Epidemiology, Social determinants

## Abstract

**Background:**

Mental illnesses contribute to a significant burden of disease in low- and middle- income countries (LMICs). Understanding risk factors for poor mental health is essential to reducing the burden, and for preventative measures to be implemented. The role of socioeconomic position (SEP) in poorer mental health is well established in high income countries, but less is known in LMICs. This study aimed to identify and synthesise epidemiological evidence for the associations between SEP and depression and suicidal ideation in LMICs in Southeast Asia, and to describe the strength and direction of any associations identified.

**Methods:**

This systematic review identified evidence by searching four databases in February 2023 (Medline, Embase, PsycInfo and Web of Science), grey literature and reference searching of included papers. Papers were included if they were based in a Southeast Asian LMIC general or hospital populations (= 16 years of age) and explored at least one measure of SEP in association with depression or suicidal ideation. Study quality was assessed using the Joanna Briggs Institute tool. A meta-analysis and narrative synthesis were performed.

**Results:**

Fifty-nine papers from six out of nine Southeast Asian LMICs were identified, with education the most commonly examined measure of SEP. Several papers explored more than one measure of SEP. Around half of the papers were rated as higher quality. Meta-analyses of education (OR: 1.87, 95% CI: 1.49-2.35) and working status (OR: 1.30, 95% CI: 0.99-1.71) provided evidence of lower levels of education and not being employed being associated with higher odds of depression. Consistent associations between lower SEP and higher odds of depression were also found for financial difficulty and subjective economic status. Three papers used suicidal ideation as their outcome, and there was some evidence of an association with lower SEP and higher ideation.

**Conclusions:**

Evidence indicates that lower SEP is associated with higher likelihood of depression, whereas for suicidal ideation, the evidence available is insufficient to reach a conclusion. The lack of longitudinal studies prevents the temporal nature of these associations being established.

**Systematic review registration:**

PROSPERO: CRD42023410444.

**Supplementary Information:**

The online version contains supplementary material available at 10.1186/s12889-024-20986-9.

## Background

Mental disorders contribute to a significant burden of disease worldwide, particularly in low- and middle- income countries (LMICs), where around 80% of those affected live [1, 2]. Of all mental disorders, depression has the greatest burden worldwide, whilst suicide, often closely associated with suicidal ideation, is one of the leading causes of mortality and years of life lost [1, 3, 4]. Understanding the risk factors for depression and suicidal ideation is important for planning and implementing interventions and preventative measures. The role of socioeconomic position (SEP) in health is well established, with lower SEP frequently associated with poorer health [5]. This association has also been observed for depression and suicidal ideation, with lower SEP associated with higher odds of depression and suicidal ideation [6, 7]. However, the majority of evidence supporting this is from high-income countries, with a paucity of evidence from LMICs.

According to the Global Burden of Disease Study, LMICs in Southeast Asia account for just over 4% of the total cases of major depressive disorder globally, however, this is likely to be an underestimate due to the way estimates are calculated [8]. A systematic review in LMICs exploring common mental disorders, including depression, found a positive relationship between poverty and common mental disorders, but the associations differed between measures of poverty [9]. Education, food insecurity, housing, social class, socio-economic status, and financial stress demonstrated relatively consistent and strong associations with common mental disorders but associations with income, employment and consumption were less consistent. However, this review included only three studies from LMICs in Southeast Asia, so it is not possible to determine if this association was replicated in this region.

There is also limited evidence, and a lack of evidence synthesis, of the association between SEP and suicidal ideation in Southeast Asia, but available evidence suggests lower levels of SEP are associated with higher odds of suicidal ideation [10–12]. Global estimates of suicide in Southeast Asia suggest that rates are low, but as with the estimates for depression, this is likely to be an underestimate due to poorer quality death records that these figures are based on [13, 14].

This systematic review aimed to synthesise the evidence for the associations between SEP and depression or suicidal ideation in LMICs in Southeast Asia, as well as describe the strength and direction of any associations identified to determine which SEP indicators were most associated with depression or suicidal ideation.

## Methods

The protocol for this study was registered with PROSPERO (CRD42023410444). This review is reported in accordance with the PRIMSA guidelines. A single study could be included more than once if they reported associations for different measures in multiple publications. For clarity, we refer to papers rather than studies.

### Searches

We searched Medline (from 1946 onwards), Embase, PsycInfo and Web of Science for articles published from 1990 onwards. Search terms were mapped to MeSH or subject headings, and used in keyword searches (see Additional files 1). Grey literature was sought through searching the World Health Organisation, the World Bank, Hinari, the World Mental Health Initiative, and the Asian Development Bank. Mental health survey reports from each country were also searched, which was an addition to that stated in the published protocol. No language restrictions were applied.

References of included papers and relevant systematic reviews identified in the search were examined for additional articles. Database and grey literature searches were completed on the 27^th^ February 2023.

### Inclusion criteria

#### Population

Papers on adults over the age of 16 were included. We used 16 as a cutoff as in Southeast Asia individuals may have finished schooling by this point, and in some countries, with parental consent get married, and are able to work in some capacity. Data had to be collected in one of the nine LMICs in Southeast Asia, defined as countries in the Association of Southeast Asian Nations [15]. This includes Cambodia, Indonesia, Laos, Malaysia, Myanmar, Philippines, Thailand, Timor Leste, and Vietnam.

Papers were not excluded based on sex, gender or age, other than already discussed Papers on school, university, and college students were excluded because attendance and availability of these institutions is likely to be socially patterned, and inclusion of these studies was likely to bias the results [16]. We excluded papers assessing depression or suicidal ideation in a specific clinical or sociodemographic population, e.g. cancer patients.

#### Exposure

The exposure was individual, household, or area-level SEP. Measures were chosen as the most common indicators of SEP in the setting they were studied, plus common measures from high-income settings [16]. The following SEP measures were included:

- Education level
- Employment
- Area-level measures of deprivation
- Deprivation
- Poverty
- Income
- Assets
- Consumption expenditure
- Aggregate/overall SEP (e.g. indices, combined measures of SEP)

We did not use search terms for the below measures but deemed them suitable measures of SEP for inclusion when subsequently identified in papers:

- Food insecurity
- Insurance ownership

Papers were subjected to full text screening if they mentioned SEP, or one of the specific SEP indicators, in the title or abstract, as an exposure variable or if they had controlled/adjusted for it.

#### Outcome

Papers measuring depression (either clinically diagnosed or self-reported symptoms) or suicidal ideation were included. Papers focussing on psychotic depression, antenatal depression, or postpartum depression (PPD) were excluded as this review was not looking at particular clinical subgroups.

#### Study designs

All observational study designs at an individual level and control arms from randomised controlled trials were included. Qualitative studies, ecological studies, case studies and reviews were excluded.

### Screening

Articles were deduplicated in Endnote then transferred to Rayyan for title and abstract screening. To ensure both screeners had the same understanding of the task, a sample of papers were assessed before completing the full screening at both title and abstract, and full text stages. Title and abstract screening were done independently by two reviewers (LB and EJ); conflicts were discussed and a decision agreed. EJ and LB agreed on 97.8% of the papers after the first screen, reaching 100% after discussions. Full text screening was completed independently by LB and BR. Google translate was used to screen one Thai language paper [17]. Conflicts were discussed and a consensus reached. A consensus was initially agreed for 73.6% of the papers, and 100% after discussions. If a paper did not provide the age range of participants included in the paper, the authors were contacted.

### Data extraction

Data were extracted using a Microsoft Access form, piloted by LB, EJ, and JD. Data on study design, participants, exposure and outcome measures and their associations were extracted by three reviewers independently; LB extracted all papers and EJ/JD performed 50% each. Where possible, associations that were not presented were calculated using raw data. If a two-by-two table contained a zero, it was not possible to calculate an association. When it could not be determined how an estimate was calculated, associations were recalculated using raw data. To compare associations across papers, the associations extracted compared the lowest SEP category to the highest, with the latter used as the reference category. Some papers did not present the associations in this way so were recalculated using the raw data or, where this was not possible, estimates were inversed.

### Quality assessment

The Joanna Briggs Institute (JBI) checklists were used to assess quality of the included papers [18]. Cross-sectional, case–control and cohort checklists were used to assess different study types. Papers were determined to be of higher quality if they met specific criteria (see Additional files 2). Criteria differed between each study type, but focused on selection, outcome definition and confounding. Regarding confounding, papers with crude (unadjusted) measures of association were deemed to be more appropriate than those who had adjusted, because of concerns about potential overadjustment, which frequently leads to biased estimates in systematic reviews of socioeconomic inequalities in health [19]. If papers did not present unadjusted estimates, an estimate was deemed overadjusted if more than sex, age, ethnicity, marital status and urbanicity had been adjusted for, as we determined that the majority of other variables would lie on the causal pathway between SEP and mental health. LB assessed all papers for quality, whilst EJ and JD did 50% each. Any discrepancies were discussed and a consensus agreed.

### Analysis

Papers were first summarised descriptively. A random effects meta-analysis was then performed if there were more than five papers rated as high quality for a given exposure and outcome, using similar measures of the exposure. Meta-analyses were performed sub-grouped by country, and pooled estimates reported when there were five or more papers. A sensitivity analysis, using forest plots and pooled analyses, included papers rated as lower quality. Where a meta-analysis was not possible, forest plots, without pooled estimates, and a narrative synthesis are presented. Only associations comparing levels of outcome across the lowest versus highest categories of SEP were shown. Crude estimates were selected over adjusted estimates.

The *metan* command was used for all analyses, performed in STATA v18. A heatmap was produced to show the number of papers by country, generated in Microsoft Excel. Findings from the papers rated as higher quality are provided in the main results section, whereas the papers rated as lower quality are presented in Additional file 3, and the sensitivity analysis in Additional file 4. We used visual inspection of funnel plots and Egger’s test to assess the possible effects of publication bias in estimates used in the meta-analysis for higher rated papers, which are shown in Additional file 4. To present the results, SEP measures were split into main and subgroup categories, which can be seen in Additional file 2.

## Results

**Fig. 1:**
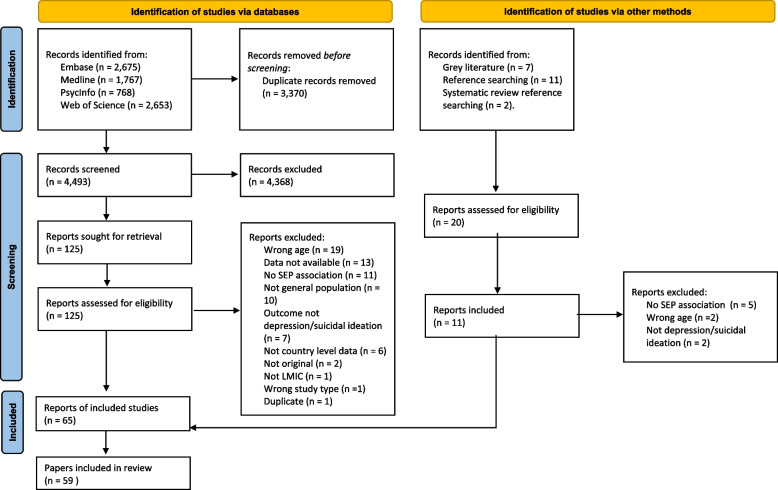
Flow chart of study selection into the systematic review

We identified 7863 articles through database searches and 20 via other methods (Fig. 1). In total 65 papers were eligible for inclusion in this review; six of these papers were duplicate reports of the same study datasets, therefore 59 papers were finally included in this review.

**Fig. 2:**
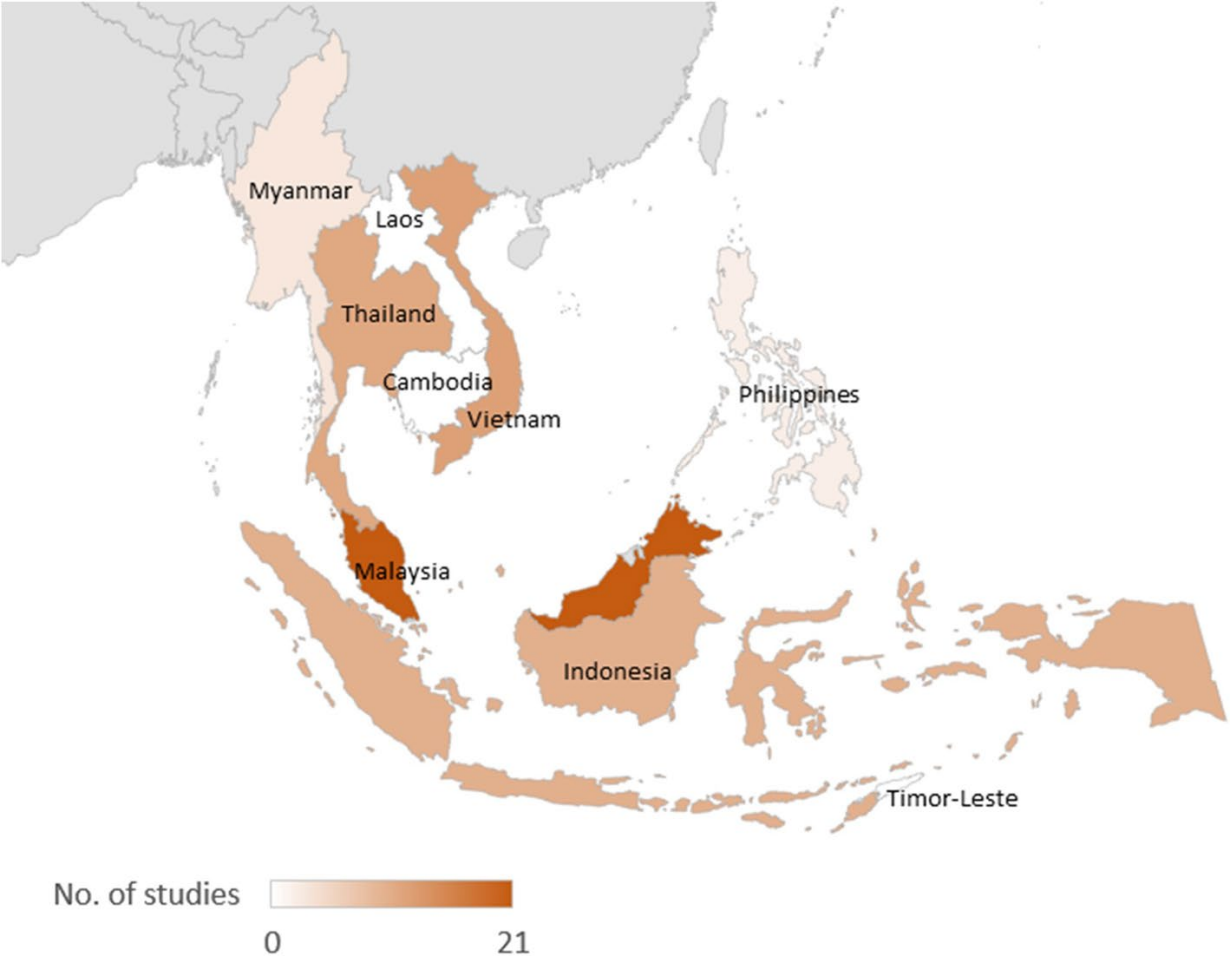
Heatmap of number of studies by country

Papers characteristics are provided in Additional files 5. The majority of papers were conducted in Malaysia (36%), and there were no eligible papers from Cambodia, Laos or Timor-Leste (Fig. 2).

Most of the papers reported on depression (95%), with three investigating suicidal ideation. The majority of papers were cross-sectional in design (86%). The most commonly measured SEP exposure was education (81%), followed by working status (employment) (51%) and then income (41%). Five papers specifically explored the association between SEP and depression during the COVID-19 pandemic [20–24]. Approximately half (54%) of the papers were restricted to the older adult/elderly population (defined differently in studies), including one paper that combined a middle-aged population with a high proportion of elderly participants [25]. Three papers were exclusively in a younger population [26–28], whilst an additional paper was entirely in a “pre-elderly” population (45–59 years old) [29]. Four papers did not state the age of their youngest participant [30–33]. One paper solely looked at associations in male participants [21].

### Quality assessment

Detailed quality ratings can be found in Additional files 2. Twenty-nine papers were rated as higher quality. None of the higher quality papers explored suicidal ideation.

### Associations of SEP with depression

#### Education

Twenty-five papers rated as higher quality explored the association between education and depression [21, 29, 34–56]. Of these, 21 were included in a meta-analysis, whilst the remaining four (which reported beta coefficients instead of odds ratios) are shown in Table 1 [21, 34–36].

**Table 1:** Associations of education and depression in papers with beta coefficients (papers rated as higher quality)

Paper	Categories	Results	Effect	Country	Sample Size
Aung (2016) (CS) [36]	Educational attainment	-0.67 (-1.08, -0.26)	Coefficient (ß) (Unadjusted)	Thailand	435
Patria. (2022) (C) [34]	Years of schooling	-0.04 (SE = 0.07)	Coefficient (ß) (Unadjusted)	Indonesia	18374
Dao (2018) (CS) [35]	University or above High school or lower	Ref 1.95 (p = 0.13)	Beta (U) (Unadjusted)	Vietnam	299
Hoang (2022) (CS) [21]	High school Less than high school	Ref 0.02 (-0.24, 0.29)	Beta (U) (Unadjusted)	Vietnam	1080

*C* Cohort, *CS* Cross-sectional, *ß* Standardised coefficient, *U* Unclear if standardised or unstandardised coefficient

**Fig. 3:**
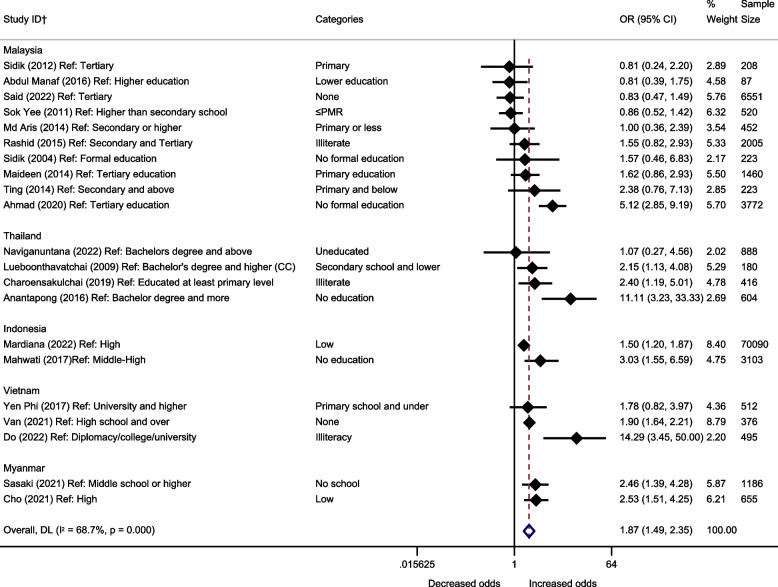
Meta-analysis of the association between education and depression (odds ratios). †All papers are cross-sectional, unless stated. CC = case-control. All estimates are unadjusted

Pooled associations (Odds Ratio (OR): 1.87, 95% CI: 1.49–2.35) (Fig. 3) of these papers suggested lower levels of education were associated with increased odds of depression, with statistical evidence to support this. There was substantial variation between estimates (I^2^ = 68.7%). There were only sufficient estimates from Malaysia for a subgroup analysis (OR: 1.37, 95% CI: 0.89–2.11, I^2^ = 70.3%), given the high I^2^ value it is unlikely that the observed heterogeneity is likely explained by country variations. Other subgroups are not reported or displayed as there weren’t sufficient estimates for pooling of results. Results from the high-quality studies not included in the meta-analysis were also consistent with an association between lower levels of education and higher depression symptoms (Table 1).

### Employment

#### Working status

**Fig. 4:**
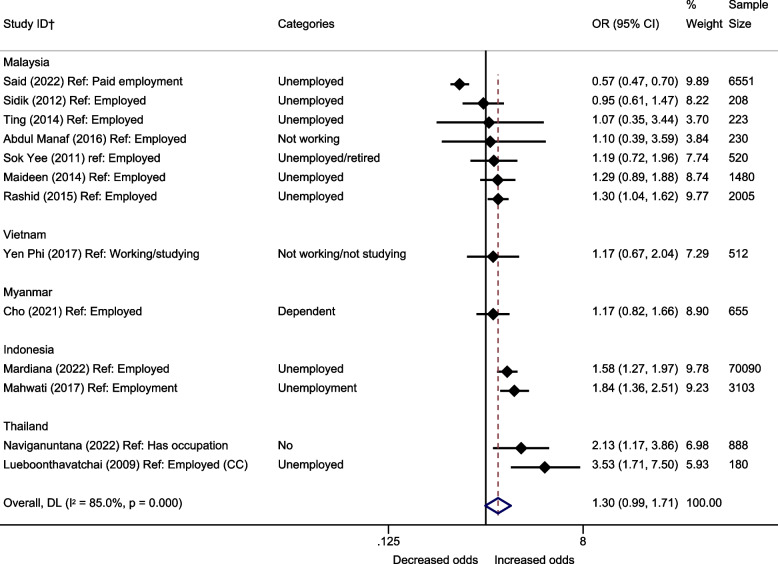
Meta-analysis of the association between working status and depression (odds ratios). †All papers are cross-sectional, unless stated. CC = case-control. All estimates are unadjusted

Working status was used as the measure of SEP in 14 papers rated as higher quality [29, 37–40, 42–45, 47, 48, 51, 52, 56]. Pooled estimates (Fig. 4) suggested weak statistical evidence that being unemployed or not working was associated with higher odds of depression compared with being in employment (OR:1.30, 95% CI: 0.99–1.71). There was substantial variation between estimates (I^2^ = 85.0%). Only Malaysia provided sufficient study estimates to be pooled, and as with education substantial heterogeneity remained between paper estimates (OR: 1.01, 95% CI: 0.70–1.46, I^2^ = 83.2%).

#### Occupation

Four papers rated as higher quality that explored occupation and depression showed limited evidence of an association (see Table 2) [21, 35, 41, 57]. Vu et al. found increased odds of depression for freelancers, compared to retired individuals, but limited evidence for other occupation groups. Md Aris et al., Hoan et al. and Dao et al. did not find strong evidence of associations with depression for a range of occupational categories compared to government workers.

**Table 2:** Associations of occupation and depression (papers rated as higher quality)

Main author (year) (study design)	Categories	Effect	Results	Country	Sample Size
Vu (2019) (CS) [57]	Retired Freelancers Farmers Others	OR (Unadjusted)	1.00 2.42 (1.01–5.62) 1.21 (0.77–1.91) 1.56 (0.71–3.32)	Vietnam	523
Md Aris (2014) (CS) [41]	Government Not working Self employed/private	OR (Unadjusted)	1.00 0.93 (0.39–2.22) 0.76 (0.36–1.68)	Malaysia	452
Hoang (2022) (CS) [21]	Medical worker Government staff Farmer Student Other	Coefficient (U) (Unadjusted)	0.091 (-0.78, 0.96) Ref -0.332 (-1.25, 0.59) 0.715 (0.29, 1.14) 0.262 (-0.13, 0.66)	Vietnam	1080
Dao (2018) (CS) [35]	Government officer Worker Business Free labour Housewife Other	Coefficient (U) (Unadjusted)	Ref 2.66 3.24 2.18 -3.64 2.82	Vietnam	299

*CS* Cross-sectional, *OR* Odds ratio, *U* Unclear if standardised or unstandardised coefficient

### Financial status

#### Income

**Fig. 5:**
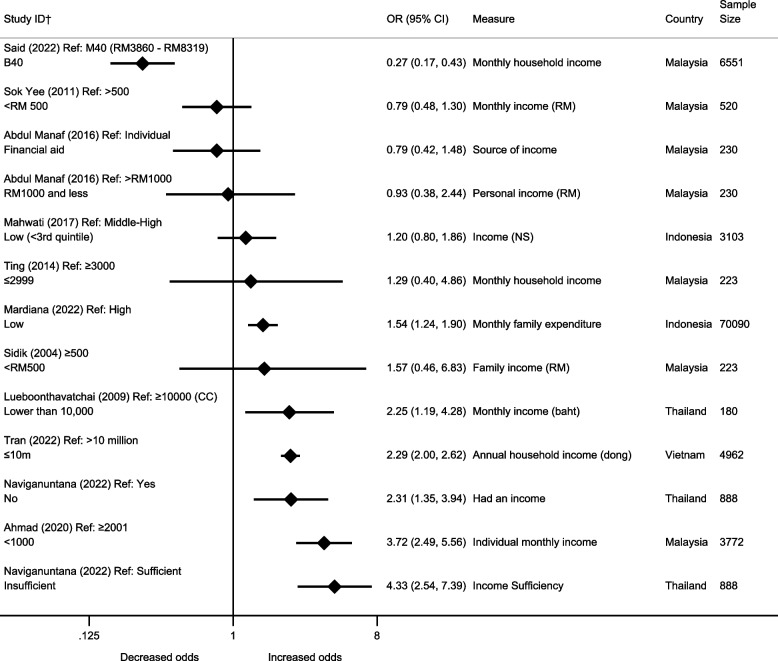
Association between income and depression in papers rated as higher quality (odds ratios). †All papers are cross-sectional, unless stated. CC=Case-Control RM = Ringgit Malaysia. All estimates are unadjusted

Eleven papers rated as higher quality assessed the association between income and depression [29, 31, 38–40, 43, 45–48, 58]. Of these, eight showed evidence of an association between lower income and increased odds of depression (Fig. 5). The use of different measures of income across the studies meant it was not appropriate to combine them in a meta-analysis. We found an increase odds of depression in those who had insufficient income, had no income, had moderate to severe financial stress and had family financial problems, however, each association was supported by just a single paper. Having lower family expenditure was also associated with increased odds of depression, backed by statistical evidence.

One paper found that a lower monthly household income was associated with a reduced odds of depression, compared to the middle group of income [39]. However, results should be interpreted with caution as the comparison group was not the highest category, as is presented in the other papers.

#### Financial difficulty

**Fig. 6:**
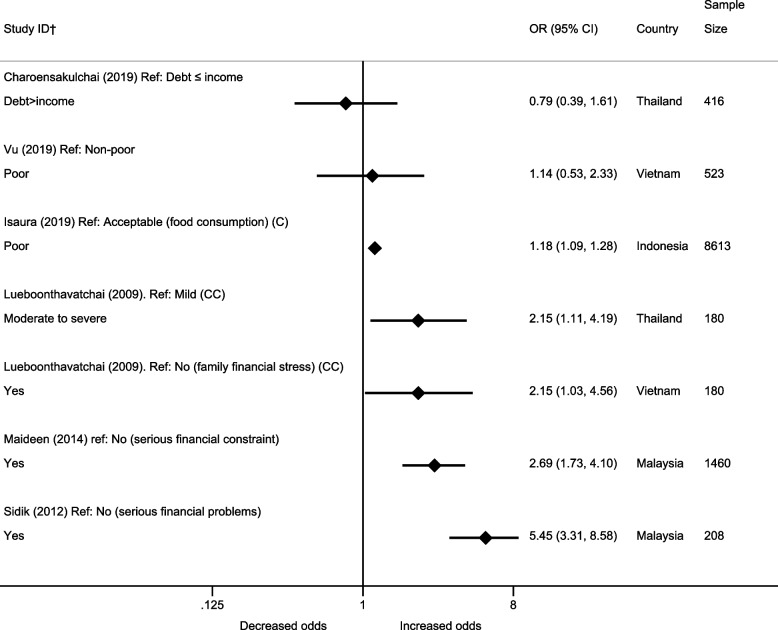
Association between financial difficulty and depression in papers rated as higher quality (odds ratios). †All papers are cross-sectional, unless stated. CC= case-control C = cohort. All estimates are unadjusted

The association between financial difficulties and depression was explored in seven papers rated as higher quality [21, 37, 44, 48, 49, 57, 59]. Five of the seven estimates suggested evidence of an association between financial difficulty and increased likelihood of depression, including two cross-sectional studies investigating serious financial problems, a case–control paper investigating family financial stress and moderate to severe financial difficulty and a cohort study investigating food consumption (Fig. 6). This cohort study also found higher food consumption score was associated with small reductions in the depressive symptom score (ß = - 9.51 × 10^–3^, 95% CI: - 6.45 × 10^-3^, - 1.26 × 10^-2^, *p* = < 0.001). Two cross-sectional studies investigating debt relative to income and being poor did not find strong evidence of association with depression.

Hoang et al. explored family financial status, according to government guidelines, and found that compared to average status, those classified as poor were more likely to experience depression with a coefficient of 1.95 (95% CI: 0.91, 2.99) [21]; comparisons were not made against the highest reference category (rich) and therefore not equivalent to other papers.

#### Subjective economic status

Two papers, rated as higher quality, explored measures of subjective economic status [52, 54]. Sasaki et al. [54] asked participants to best describe their current financial situation in light of general economic conditions [54]. The results suggest that those who see themselves as having difficult or very difficult financial situations had increased odds of having depression (crude OR (cOR): 4.82, 95% CI: 3.51–6.62). Yen Phi et al. (2017) demonstrated similar results in a Vietnamese sample, recruited from a primary care setting (cOR: 8.54, 95% CI: 2.35–30.98) [52].

### Asset based

#### Composite

Two papers rated as higher quality used a composite measure as their SEP exposure [54, 60]. Composite measures are defined if the authors used more than one variable to create the measure (Table 3).

**Table 3:** Composite measures as socioeconomic position exposure

Main author (year)	Measure	Derived
Sasaki (2021) (54)	Wealth Index	Ownership of: Radio, black & white television, colour television, Video/DVD player, electric fan, refrigerator, computer, store-bought furniture, personal music player, washing machine, gas cooker, electric cooker or rice cooker, air conditioner, bicycle, motorcycle, van/truck, microwave oven, mobile telephone, and internet
Jittawisuthikul (2011) (60)	Socioeconomic status	Household assets (not stated)

Sasaki et al. found evidence that those with a low wealth index had increased odds of depression (cOR: 1.88, 95% CI: 1.40–2.50) compared to middle/high wealth [54]. One longitudinal paper reported on an aggregate measure of SEP, derived from number of household assets owned [60]. The measure was then classified as high or low depending on whether an individual’s index scores were above or below the median index score. Those classified in the low category had a 22% reduced risk of depression,- compared to those classified in the high category (95% CI: 0.40–1.17).

#### Ownership

Two papers rated as higher quality reported on ownership of single assets [29, 57]. They found weak evidence of an association between not owning insurance and a reduced odds of depression (cOR: 0.86, 95% CI: 0.92–1.19) as well as limited evidence of not owning health insurance and an increased odds of depression (cOR: 1.13, 95% CI: 0.64–2.07).

Comparison between low and high quality papers

### Comparison between low and high quality papers

Results of the sensitivity analysis with education and working status can be seen in Additional files 4, and show consistency with results from papers rated as higher quality. Inclusion of the papers rated as lower quality substantially increased the heterogeneity in the education analyses (I^2^ = 80.5%).

Results for subjective economic status, ownership, income, and occupation were similar, regardless of the rated quality of the study. Asset based measures were more strongly positively associated with depression in papers rated as lower quality, compared to those rated as higher. The association between depression and financial difficulty was weaker in papers rated as lower quality, with direction of associations remaining the same, but wider confidence intervals that mostly crossed the null.

Three papers rated lower quality explored the association between SEP and suicidal ideation (Additional files 3), and found evidence of an association between lower levels of education and high odds of depression. Working status, income and financial difficulty were only explored in one paper, and broadly found associations between lower levels of SEP and higher levels of ideation.

## Discussion

### Main findings

Fifty-six papers were identified that explored at least one measure of SEP in association with depression, in six out of nine Southeast Asian LMICs. The majority of included studies were conducted in Malaysia [23, 31, 37–46, 61–69]. A small number of papers identified explored the association between measures of SEP and suicidal ideation (*n* = 3) [26, 62, 70].

The association between lower levels of SEP and higher likelihood of depression were most consistent with education, working status, financial difficulty, and subjective economic status. Occupation, income, composite measures, and ownership showed inconsistent findings, with variable results in both the strength and direction of associations. The inconsistencies in findings may be due to the variations in how these SEP variables were defined, and contextual differences between the countries or the reference category used.

Three papers rated as lower quality explored the association of SEP with suicidal ideation, and whilst the direction of association was consistent when exploring education (i.e. lower education was associated with higher odds of suicidal ideation), there was not always statistical evidence to support this, potentially representing underpowered studies. Only one of these studies included suicidal ideation as the primary outcome (for which weak evidence of an association was found), which was also the only study to conduct a sample size calculation [70].

### Comparison with other studies-depression

A recent systematic review explored the association between socioeconomic status and depression, considering only composite/asset-based measures of SEP [71]. The authors concluded that the lower the socioeconomic status, the higher the risk of depression. They also found that the strength of associations with subjective measures of SEP were greater than objective measures; our results also found consistent positive results with subjective economic status. Subjective measures are often considered to be more closely related to the perceived position in society, compared to objective measures, and may play a more important role in the associations with depression through stress and feeling unable to provide as required [72]. The review did not include any papers we identified, possibly due to our review including papers where SEP was a covariate, nor did it include any papers from Southeast Asia with the majority of papers coming from the United States and China. An older meta-analysis also explored the associations between socioeconomic inequalities and prevalence or incidence depression [6]. As above, there were no papers from Southeast Asia included but there was more diversity of countries (24 included versus 10 above) in the studies included. Results suggest that there are socioeconomic inequalities in incidence and prevalence of depression, with higher SEP groups at lowest likelihood of depression, which was broadly replicated in our findings.

Another review conducted mostly in high-income settings looking at financial indicators found a positive association between financial hardship, and subjective financial strain, with depression, in line with our conclusions [73]. Guan *et al. *found that household assets were a strong indicator of depression, but we identified mixed evidence surrounding this measure in high quality papers; in lower quality papers, our results are similar to Guan et al. This may be due to the differences in assets used and the importance of them in different contexts, or could be due to the use of adjusted estimates, and therefore could suggest overadjustment by other sociodemographic variables [16]. Guan et al. also found mixed evidence for income, suggesting that how income was measured affected the strength and direction of association, which is a plausible reason for the inconsistencies in our review, which included multiple definitions of income across studies. It is also possible that some of our inconsistencies in the associations with income are due to a high proportion of the papers being conducted in older populations, although we did not investigate this as this was not the focus of this review.

It is likely that associations with working status, occupation and income may be closely linked. No papers in our review explore the interactions between these variables. For example, although those who are working are perceived to have higher socioeconomic status than those who are unemployed, there are many different types of working status e.g. self-employed, which may in itself be a precarious situation due to uncertainties over income, subsequently resulting in higher odds of depression [74]. The aim of this review was not to disentangle these relationships but to acknowledge that they do exist. It is also important to consider when the data were collected as contextual issues e.g. an economic crisis, COVID-19, severe weather event may contribute towards increased levels of depression that are interlinked to occupation, income and employment, and external contributing factors towards depression [75–77].

Within our review, the papers included used a wide variety of tools to assess depression. The differences in outcome measurement could explain some of the heterogeneity seen in the meta-analysis results for education and working status. Papers rated as higher quality used tools that had been validated previously, however, this was not always in the setting it was then subsequently used. Stigma is often attached to mental health disorders in Southeast Asia, including the presence of depression [78, 79]. Using tools that are not validated in the setting it is used may mean that depression was underreported in the papers we included, as they may ask questions not deemed culturally sensitive i.e. participants may be more willing to report physical symptoms than psychological ones. Similarly, using cut-offs of depression that are meant for different settings could also lead to inaccurate estimates [80].

### Comparisons with other studies—suicidal ideation

To the best of our knowledge, there are no other systematic reviews exploring the association between SEP and suicidal ideation in Southeast Asia. However, there was a review looking at the relationship in South Korea (a high income country) [7]. This study was similar in that it explored multiple socioeconomic indicators in relation to suicidal ideation. For education, their findings differ slightly from what we identified. Although the majority of their papers showed a positive association between lower education and suicidal ideation, they had additional studies that demonstrated the reverse. The South Korean review found that, in some of their included studies, income had a greater effect on men than women, which was consistent with findings in our review. However, our review had reverse findings when comparing results for different age categories, with lower income being associated with lower odds of ideation, albeit not supported by statistical evidence. It is important to note that we only included one study exploring income in association with suicidal ideation, and therefore it is difficult to draw comparisons to a review that included 15.

There are limited systematic reviews to compare our results to that explore suicidal ideation. However, a systematic review was conducted on suicide and attempted suicide, both related to suicidal ideation, in South and Southeast Asia that explored SEP [81]. Despite a different outcome, this review also found mixed evidence regarding income, but found more consistent evidence of an association with education.

### Underlying mechanisms

The theories behind the mechanisms of the relationships between SEP and depression/suicidal ideation are broadly divided into two hypotheses: social causation and social selection/drift. The former suggests that the conditions of poverty, such as increased stressors, limited access to health care and increased traumatic life events, are thought to increase poor mental health. This may be supported by our evidence that suggests education, a measure often attained earlier in life, is associated with higher odds of depression and ideation. Lower education levels may lead to poorer or more limited job opportunities, resulting in reduced income and therefore additional stressors which can increase poor mental health. The latter theory, social selection/drift, suggests the inverse: poor mental health results in increased conditions of poverty due to loss of employment and subsequently reduced income, as examples. Most of the literature concentrates on the social causation hypothesis in relation to depression, particularly when looking at associations longitudinally, although recent evidence from South Africa indicates that the causal mechanism also involves social drift [82]. Given the vast majority of findings and basis for these theories arise from studies in high-income countries, it cannot be assumed that these will also apply to LMICs. Lower SEP is likely to be associated with more acute levels of poverty and access to mental health services considerably more limited in LMICs compared to high-income countries, which may mean that associations between SEP and mental health due to social causation are stronger in LMICs. However, higher mental health stigma in LMIC compared to high-income countries could also lead to stronger associations due to social section/drift. High quality longitudinal studies are needed to investigate these theories in the Southeast Asian setting.

### Strengths and limitations

This review employed a comprehensive search strategy which identified relevant papers which have been missed from existing systematic review on this topic [9, 71, 73, 83]. This review did not restrict on language and also searched grey literature. The review does, however, have some limitations. Firstly, whilst efforts were made to include papers that mentioned SEP in their title or abstract, we did not explore all papers to see if the authors had included SEP as a confounder in their analyses but had not mentioned it in their abstract. We may have missed eligible papers because of this. However, previous work suggests this is likely to have omitted a minimal number of studies [84].

Additionally, the assessment of quality did not take into account response rates therefore we may have incorrectly rated a study as being low risk of bias (i.e. higher quality) when the results were impacted by a non-response bias. Many of the papers did not report response bias so this review could not reliably assess that. Relatedly higher quality papers had to have a crude estimate of association, to limit the potential for overadjustment of associations, a common problem in systematic reviews of socioeconomic inequalities [19]. It is possible that under adjustment for some measures e.g sex may be present. Finally, excluding papers exploring PPD/maternal depression may have resulted in a large female population being missed. However, inclusion may have biased the results by including a more clinical population than other types of study and by collider bias due to socioeconomic differences in birth rates.

## Conclusions

We found evidence that lower SEP increases the likelihood of depression in Southeast Asian LMICs, with some indication of this association with suicidal ideation. Education, working status, financial status and subjective economic status show the most consistent associations between lower SEP and increased likelihood of depression. Our review highlights the need for additional research exploring SEP in relation to depression and suicidal ideation in all LMIC in Southeast Asia. Similarly, more work is needed to ensure tools used for identification of poor mental health are validated in the relevant settings before use of them in epidemiological studies. Further investigations into whether the relationships we have examined are causal would aid political decision-making surrounding interventions and funding needs.

## Supplementary Information

Supplementary Material 1.

Supplementary Material 2.

Supplementary Material 3.

Supplementary Material 4.

Supplementary Material 5.

## Data Availability

All data generated or analysed during this study are included in this published article and its supplementary information files.

## Abbreviations

LMICs: Low- and middle- income countries
PPD: Post partum depression
SEP: Socioeconomic position
