# Supplementary material for: The association between socioeconomic position and depression or suicidal ideation in low- and middle-income countries in Southeast Asia: a systematic review and meta-analysis
Source: BMC Public Health. 2024 Dec 18;24:3507. doi: 10.1186/s12889-024-20986-9 (PMC11656959; doi:10.1186/s12889-024-20986-9)
Supplement: Supplementary file 1 — Supplementary Material 1. [file 12889_2024_20986_MOESM1_ESM.docx]

# Additional files 1

**Medline**

1. Exp Socioeconomic Factors/ or Education/ or Occupations/ or unemployment/ or employment/ or Income/ or exp Poverty/ or economics/ or crowding/ or Social deprivation/ or exp Social Class/ or exp Health Inequities/ or exp economic factors/
2. (socioeconomic or socio economic or education* or Occupation or Employ* or Unemploy* or Income or Job or Money or Social* or social class or Poverty or Finance or Wealth or Culture or ethnolog* or Depriv* or bankrupt* or economic or consumption expenditure or debt or rural or urban or (car adj2 ownership) or (vehicle adj2 ownership) or (motor* adj2 ownership) or (bike adj2 ownership) or (television adj2 ownership) or (tv adj2 ownership) or (internet adj2 access) or (phone adj2 ownership) or neighborhood or neighbourhood or inequalit* or slum or population density or disadvantage* or impoverish* or household construction or household material or electricity supply or housing instability or (home adj2 ownership)).kw,ti,ab.
3. Depression/ or Suicidal Ideation/
4. (Depress* or suicidal ideation or suicidal thought* or suicidal).ti,ab,kw.
5. Cambodia/ or Indonesia/ or Laos/ or Malaysia/ or Myanmar/ or Philippines/ or Thailand/ or Vietnam/ or Timor-Leste/
6. (asia or Cambodia or Indonesia or Lao or Laos or Malaysia or Myanmar or Philippines or Thailand or Vietnam or Timor-Leste or Timor Leste or TimorLeste or east timor or east-timor or easttimor or west timor or west-timor or westtimor or Burma).ti,ab,kw.
7. 1 or 2
8. 3 or 4
9. 5 or 6
10. 7 and 8 and 9
11. Limit 10 to yr=”1990- Current”
12. (qualitative not quantitative).ti.
13. (("u.s." and miliarty) or (us and military) or (us and soldiers) or ("u.s." and soldiers) or vietnam war).ti,ab,kw.
14. ("book review" or "conference proceeding review" or "journal conference review" or "journal review" or "report review" or "review" or "comment" or case reports).pt.
15. 11 not (12 or 13 or 14)

**PsychInfo**

1. Exp socioeconomic factors/ or exp Socioeconomic Status/ or exp Social Class/ or exp Family Socioeconomic Level/ or exp Educational Background/ or education/ or exp Income level/ or exp occupations/ or exp employment status/ or exp poverty/ or poverty areas/ or exp finance/ or money/ or Deprivation/or exp social deprivation/ or financial strain/ or economics/ or health disparities/ or crowding/
2. (socioeconomic or socio economic or education* or Occupation or Employ* or Unemploy* or Income or Job or Money or Social* or social class or Poverty or Finance or Wealth or Culture or ethnolog* or Depriv* or bankrupt* or economic or consumption expenditure or debt or rural or urban or (car adj2 ownership) or (vehicle adj2 ownership) or (motor* adj2 ownership) or (bike adj2 ownership) or (television adj2 ownership) or (tv adj2 ownership) or (internet adj2 access) or (phone adj2 ownership) or neighborhood or neighbourhood or inequalit* or slum or population density or disadvantage* or impoverish* or household construction or household material or electricity supply or housing instability or (home adj2 ownership)).ti,ab.
3. Suicidal ideation/ or depression/ or major depression/
4. (Depress* or suicidal ideation or suicidal thought* or suicidal).ti,ab.
5. (asia or Cambodia or Indonesia or Lao or Laos or Malaysia or Myanmar or Philippines or Thailand or Vietnam or Timor-Leste or Timor Leste or TimorLeste or east timor or east-timor or easttimor or west timor or west-timor or westtimor or Burma).ti,ab.
6. 1 or 2
7. 3 or 4
8. 6 and 7 and 8
9. Limit 8 to yr=”1990-Current”
10. (Non-peer-reviewed-journal or book or authored book or edited book or encyclopedia or dissertation abstract).pt.
11. (("u.s." and miliarty) or (us and military) or (us and soldiers) or ("u.s." and soldiers) or vietnam war).ti,ab.
12. (qualitative not quantitative).ti.
13. 9 not (10 or 11 or 12)

**Embase**

1. exp socioeconomics/ or Education/ or Occupation/ or unemployment/ or exp employment/ or Income/ or exp household income/ or exp Lowest income group/ or exp Poverty/ or poverty level/ or Social Class/ or exp Health disparity/
2. (socioeconomic or socio economic or education* or Occupation or Employ* or Unemploy* or Income or Job or Money or Social* or social class or Poverty or Finance or Wealth or Culture or ethnolog* or Depriv* or bankrupt* or economic or consumption expenditure or debt or rural or urban or (car adj2 ownership) or (vehicle adj2 ownership) or (motor* adj2 ownership) or (bike adj2 ownership) or (television adj2 ownership) or (tv adj2 ownership) or (internet adj2 access) or (phone adj2 ownership) or neighborhood or neighbourhood or inequalit* or slum or population density or disadvantage* or impoverish* or household construction or household material or electricity supply or housing instability or (home adj2 ownership)).kw,ti,ab.
3. Depression/ or Suicidal ideation/
4. (Depress* or suicidal ideation or suicidal thought* or suicidal).ti,ab,kw.
5. Cambodia/ or exp Indonesia/ or Laos/ or exp Malaysia/ or Myanmar/ or Philippines/ or Thailand/ or Viet Nam/ or Timor-Leste/
6. (asia or Cambodia or Indonesia or Lao or Laos or Malaysia or Myanmar or Philippines or Thailand or Vietnam or Timor-Leste or Timor Leste or TimorLeste or east timor or east-timor or easttimor or west timor or west-timor or westtimor or Burma).ti,ab,kw.
7. 1 or 2
8. 3 or 4
9. 5 or 6
10. 7 and 8 and 9
11. Limit 10 to yr="1990 -Current"
12. (("u.s." and miliarty) or (us and military) or (us and soldiers) or ("u.s." and soldiers) or vietnam war).ti,ab,kw.
13. (qualitative not quantitative).ti.
14. ("book review" or "conference proceeding review" or "journal conference review" or "journal review" or "report review" or "review" or "preprint" or "letter" or "editorial" or "note" or "data paper" or editorial or erratum).pt.
15. 11 not (12 or 13 or 14)

**Web of Science**

1. (TS=(socioeconomic or socio economic or education* or Occupation or Employ* or Unemploy* or Income or Job or Money or Social* or social class or Poverty or Finance or Wealth or Culture or ethnolog* or Depriv* or bankrupt* or economic or consumption expenditure or debt or rural or urban or neighborhood or neighbourhood or inequalit* or slum or population density or disadvantage* or impoverish* or household construction or household material or electricity supply or housing instability) AND TS=(depression or suicidal or suicidal ideation or suicidal thought*) AND TS=(asia or Cambodia or Indonesia or Lao or Laos or Malaysia or Myanmar or Philippines or Thailand or Vietnam or Timor-Leste or Timor Leste or timorese or east timor or east-timor or elasttimer or west timor or west-timor or westmar or Burma))
2. Limit publication date 01/01/1990 – 27/02/2023
