# Supplementary material for: The association between socioeconomic position and depression or suicidal ideation in low- and middle-income countries in Southeast Asia: a systematic review and meta-analysis
Source: BMC Public Health. 2024 Dec 18;24:3507. doi: 10.1186/s12889-024-20986-9 (PMC11656959; doi:10.1186/s12889-024-20986-9)
Supplement: Supplementary file 2 — Supplementary Material 2. [file 12889_2024_20986_MOESM2_ESM.docx]

# Additional files 2

Table 1. Questions used to determine higher quality studies from the Joanna Briggs Institute checklists

| **Study design** | **Questions from the overall scale number** | **Criteria to be met** |
| --- | --- | --- |
| Case-control | 1 | Groups were comparable other than the presence of disease in cases or the absence of disease in controls |
|  | 7 | Strategies to deal with confounding factors were stated |
|  | 8 | Depression/suicidal ideation was measured in a valid and reliable way i.e for depression, a validated tool, or clinical diagnosis, was appropriate, but a single question asking “are you depressed”, was not. For suicidal ideation, a single question was fine. |
| Cohort study | 1 | Two groups were similar and recruited from the same population |
|  | 2 | Exposures were measured similarly to assign exposure status |
|  | 5 | Strategies to deal with confounding factors were stated |
|  | 7 | Depression/suicidal ideation was measured in a valid and reliable way i.e for depression, a validated tool, or clinical diagnosis, was appropriate, but a single question asking “are you depressed”, was not. For suicidal ideation, a single question was fine. |
| Cross sectional | 1 | Criteria for inclusion was clearly defined |
|  | 2 | Study participants and setting were described in detail |
|  | 6 | Strategies to deal with confounding factors were stated |
|  | 7 | Depression/suicidal ideation was measured in a valid and reliable way i.e for depression, a validated tool, or clinical diagnosis, was appropriate, but a single question asking “are you depressed”, was not. For suicidal ideation, a single question was fine. |

Table 2. JBI quality scores for cohort studies. ^[[1]](#footnote-1)^

| **Cohort** | *Fakhrunnisak (2022) (68)* | *Isaura (2019) (69)* | *Jittawisuthikul (2011) (70)* | *Kim (2020) (71)* | *Patria (2022) (72)* | *Peltzer (2022) (73)* |
| --- | --- | --- | --- | --- | --- | --- |
| Q1 | Yes | Yes | Yes | Yes | Yes | Yes |
| Q2 | Yes | Yes | Yes | Yes | Yes | Yes |
| Q3 | No | Yes | Yes | Yes | No | Yes |
| Q4 | No | Yes | Yes | Yes | No | Yes |
| Q5 | Unclear | Yes | Yes | No | Yes | No |
| Q6 | No | Unclear | Yes | Yes | No | No |
| Q7 | Yes | Yes | Yes | No | Yes | Yes |
| Q8 | Yes | Yes | No | Yes | Yes | Unclear |
| Q9 | No | No | Yes | No | No | No |
| Q10 | No | No | No | No | No | No |
| Q11 | No | Yes | Yes | Yes | Unclear | Yes |
| Quality | Low | High | High | Low | High | Low |

Table 3. JBI quality scores for case-control studies.^[[2]](#footnote-2)^

| **Case-control** | *Lueboonthavatchai (2009) (74)* | *Mumang (2020) (75)* |
| --- | --- | --- |
| Q1 | Yes | No |
| Q2 | No | No |
| Q3 | Yes | No |
| Q4 | Yes | Yes |
| Q5 | Yes | Yes |
| Q6 | Yes | Yes |
| Q7 | Yes | Yes |
| Q8 | Yes | Yes |
| Q9 | Yes | Yes |
| Q10 | Yes | Yes |
| Quality | High | Low |

| **Cross Sectional** | *Abdul Manaf (2016) (19)* | *Ahmad (2020) (20)* | *Anantapong (2016) (21)* | *Aung (2016) (22)* | *Charoensakulchai (2019) (23)* | *Cheah (2018) (24)* | *Cheah (2019) (25)* | *Cheung (2009) (26)* | *Cho (2021) (27)* | *Collier (2020) (28)* | *Dao (2018) (29)* | *Do (2022) (30)* | *Duong (2020) (31)* | *Foong (2021) (32)* | *Giang (2019) (33)* | *Haseen (2011) (34)* | *Hoang (2022) (35)* | *Idaiani (2021) (36)* | *Imran (2009) (37)* | *Kim (2020) (38)* | *Leggett (2012) (39)* | *Madyaningrum (2019) (40)* | *Mahwati (2017) (41)* | *Maideen (2014) (42)* | *Manaf (2016) (43)* |  |
| --- | --- | --- | --- | --- | --- | --- | --- | --- | --- | --- | --- | --- | --- | --- | --- | --- | --- | --- | --- | --- | --- | --- | --- | --- | --- | --- |
| Q1 | Yes | Yes | Yes | Yes | Yes | No | Yes | Yes | Yes | No | Yes | Yes | Yes | No | Yes | No | Yes | Yes | Yes | No | Yes | Yes | Yes | Yes | No |  |
| Q2 | Yes | Yes | Yes | Yes | Yes | Yes | Yes | Yes | Yes | Yes | Yes | Yes | No | Yes | Yes | No | Yes | Yes | Yes | Yes | Yes | Yes | Yes | Yes | Yes |  |
| Q3 | Yes | Yes | Yes | Yes | No | Yes | Yes | Yes | Yes | Yes | Yes | No | Yes | Yes | Yes | Yes | Yes | Yes | Yes | Yes | No | Yes | Yes | No | Yes |  |
| Q4 | Yes | Yes | Yes | Yes | Yes | Yes | No | Yes | Yes | Yes | Yes | Yes | Yes | Yes | No | Yes | Yes | Yes | Yes | Yes | Yes | Yes | Yes | Yes | Yes |  |
| Q5 | Yes | Yes | Yes | Yes | Yes | Yes | Yes | Yes | Yes | Yes | Yes | Yes | Yes | Yes | Yes | Yes | Yes | Yes | No | Yes | Yes | Yes | Yes | Yes | Yes |  |
| Q6 | Yes | Yes | Yes | Yes | Yes | Yes | No | No | Yes | Yes | Yes | Yes | Yes | No | No | No | Yes | No | No | No | No | No | Yes | Yes | No |  |
| Q7 | Yes | Yes | Yes | Yes | Yes | Yes | No | Yes | Yes | Yes | Yes | Yes | Yes | Yes | No | Yes | Yes | Yes | Yes | Yes | Yes | Yes | Yes | Yes | Yes |  |
| Q8 | Yes | Yes | Yes | Yes | Yes | Yes | Yes | Yes | Yes | Yes | Yes | Yes | Yes | Yes | Unclear | Yes | Yes | Yes | No | Yes | Yes | Yes | Yes | Yes | Yes |  |
| Quality | High | High | High | High | High | Low | Low | Low | High | Low | High | High | Low | Low | Low | Low | High | Low | Low | Low | Low | Low | High | High | Low |  |
|  | *Mardiana (2022) (44)* | *Md Aris (2014) (45)* | *Mesbah (2020) (46)* | *Mubasyiroh (2022) (47)* | *Naviganuntana (2022) (48)* | *Peltzer (2022) (49)* | *Prueksaritanond (2007) (50)* | *Rashid (2015) (51)* | *Razali (2022) (52)* | *Said (2022) (53)* | *Sasaki (2021) (54)* | *Sharma (2021) (55)* | *Sherina (2004) (15)* | *Sidik (2003) (56)* | *Sidik (2004) (16)* | *Sidik (2012) (57)* | *Sok Yee (2011) (58)* | *Ting (2014) (59)* | *Tran (2022) (60)* | *Van (2021) (61)* | *Vanoh (2016) (62)* | *Vu (2019) (63)* | *Wichaidit (2022) (64)* | *Yamada (2019) (65)* | *Yen Phi (2017) (66)* | *Yeoh (2017) (67)* |
| Q1 | Yes | Yes | Yes | No | Yes | Yes | Yes | Yes | Yes | Yes | Yes | No | Yes | Yes | Yes | Yes | Yes | Yes | Yes | Yes | Yes | Yes | Yes | Yes | Yes | No |
| Q2 | Yes | Yes | No | No | Yes | Yes | No | Yes | No | Yes | Yes | Yes | No | Yes | Yes | Yes | Yes | Yes | Yes | Yes | Yes | Yes | Yes | Yes | Yes | No |
| Q3 | Yes | Yes | Yes | Yes | Yes | No | Unclear | Yes | Yes | Yes | Yes | Yes | Yes | Yes | Yes | No | Yes | Yes | Yes | Yes | No | Yes | Yes | Yes | Yes | Yes |
| Q4 | Yes | Yes | Yes | Yes | Yes | Yes | Yes | Yes | Yes | No | Yes | Yes | Yes | Yes | Yes | Yes | Yes | Yes | Yes | Yes | Yes | Yes | Yes | Yes | Yes | Yes |
| Q5 | Yes | Yes | Yes | Yes | Yes | Yes | Unclear | No | Yes | Yes | Yes | Yes | No | Yes | No | Yes | No | Yes | Yes | Yes | No | Yes | Yes | Yes | Yes | Yes |
| Q6 | Yes | Yes | Yes | No | Yes | No | Yes | Yes | Yes | Yes | Yes | No | No | No | Yes | Yes | Yes | Yes | Yes | No | No | Yes | No | No | Yes | No |
| Q7 | Yes | Yes | Yes | Yes | Yes | Yes | Yes | Yes | Yes | Yes | Yes | Yes | Yes | Yes | Yes | Yes | Yes | Yes | Yes | Yes | Yes | Yes | Yes | Yes | Yes | Yes |
| Q8 | Yes | Yes | Yes | Yes | Yes | Yes | Unclear | Yes | No | Yes | Yes | Yes | No | No | No | No | No | No | Yes | Yes | No | Yes | Yes | Unclear | Yes | Yes |
| Quality | High | High | Low | Low | High | Low | Low | High | Low | High | High | Low | Low | Low | High | High | High | High | High | Low | Low | High | Low | Low | High | Low |

Table 4. JBI quality scores for cross sectional studies^[[3]](#footnote-3)^

Table S5. SEP groupings and measurements

| **Main Group** | **Subgroup** | **SEP Measurement** |
| --- | --- | --- |
| Education | Educational level | Highest qualification |
|  |  | Years of education |
|  |  | Parental education |
|  |  | No formal education |
|  |  | Literacy |
| Employment | Occupation | Occupation type |
|  | Working status | Employment status |
| Financial Measures | Income | Personal monthly income |
|  |  | Family/household annual income |
|  |  | Personal annual income |
|  |  | No income generated |
|  |  | Income sufficiency |
|  |  | Source of income |
|  |  | Family/household monthly income |
|  |  | Expenditure |
|  | Subjective economic status | Self-rated financial status |
|  | Financial difficulty | Debt |
|  |  | Financial stress |
|  |  | Food insecurity |
|  |  | Economic dependence |
|  |  | Financial/poverty status |
| Asset based measures | Composite measures | Wealth index |
|  |  | Asset score |
|  |  | Value of household assets |
|  |  | Multidimensional summary measure |
|  | Ownership/access | Land ownership |
|  |  | House ownership |
|  |  | Insurance ownership |
|  |  | Indoor water supply |
|  |  | Indoor kitchen |

1. Q1= Were the two groups similar and recruited from the same population? Q2 = Were the exposures measured similarly to assign people to both exposed and unexposed groups? Q3 = Was the exposure measured in a valid and reliable way? Q4 = Were confounding factors identified? Q5 = Were strategies to deal with confounding factors stated? Q6 = Were the groups/participants free of outcome at the start of the study? Q7 = Were the outcomes measured in a valid and reliable way? Q8 = Was the follow up time reported and sufficient to be long enough for outcomes to occur? Q9 = Was follow up complete, and if not, were the reasons to loss to follow up described and explored? Q10 = Were the strategies to address incomplete follow up utilized? Q11 = Was appropriate statistical analysis used? [↑](#footnote-ref-1)
2. Q1 = Were the groups comparable other than the presence of disease in cases of the absence of disease in controls? Q2 = Were the cases and controls matched appropriately? Q3 = Were the same criteria used for identification of cases and controls? Q4 = Was exposure measured in a standard, valid and reliable way? Q5 = Was exposure measured in the same way for cases and controls? Q6 = Were confounding factors identified? Q7 = Were the strategies to deal with confounding factors stated? Q8 = Were the outcomes assessed in a standard, valid and reliable way for cases and controls? Q9 = Was the exposure period of interest long enough to be meaningful? Q10 = Was appropriate statistical analysis used? [↑](#footnote-ref-2)
3. *Q1 = 1. Were the criteria for inclusion in the sample clearly defined? Q2 = Were the study subjects and the setting described in detail? Q3 = Was the exposure measured in a valid and reliable way? Q4 = Were objective, standard criteria used for measurement of the condition? Q5 = Were confounding factors identified? Q6 = Were strategies to deal with confounding factors stated? Q7 = Were the outcomes measured in a valid and reliable way? Q8 = Was appropriate statistical analysis used?* [↑](#footnote-ref-3)
