# Supplementary material for: The association between socioeconomic position and depression or suicidal ideation in low- and middle-income countries in Southeast Asia: a systematic review and meta-analysis
Source: BMC Public Health. 2024 Dec 18;24:3507. doi: 10.1186/s12889-024-20986-9 (PMC11656959; doi:10.1186/s12889-024-20986-9)
Supplement: Supplementary file 3 — Supplementary Material 3. [file 12889_2024_20986_MOESM3_ESM.docx]

# Additional files 3

## Lower Quality Papers

The following section provides findings for the associations between SEP and depression/suicidal ideation from papers rated as lower quality.

## Associations of SEP with depression

Table 1 presents which variables estimates have been adjusted for, if crude ratios were not present, for depression as the outcome. Where crude ratios were available, they have been presented and are not included in the table.

Table 1. Variables that have been controlled for in papers that only have adjusted estimates
C = cohort, SEP = socioeconomic position, SES = socioeconomic status, U = unclear, X = adjusted for, - = not adjusted for

| **Main author (year)** | **Sex** | **Age** | **Marital Status** | **Urban/rural or geographic area** | **Smoking** | **Ethnicity** | **Other SEP** | **Mental illness** | **Physical Health** | **Other** | **Measures of SEP** |
| --- | --- | --- | --- | --- | --- | --- | --- | --- | --- | --- | --- |
| *Cheah (2019) (1)* | X | X | X | - | X | X | SEP exposures measured | - | X | Physical activity, family size | Education  Income Work status |
| *Fakhrunnisak (2022) (C) (2)* | U | U | U | U | U | U | U | U | U | U | Parental education |
| *Foong (3)* | X | X | X | - | - | - | SEP exposures measured | - | - | Living arrangements | Education Income  Work status |
| *Giang (2019) (4)* | X | X | X | - | - | - | SEP exposures measured | - | X | Living arrangements, experienced domestic violence, had a role in family decisions, received help from children, Took care of (great)grandchild(ren) or other family members, Participated in social organizations and community activities, had respect from community as an older person. | Education Financial difficulty  Work status |
| *Haseen (2011) (5)* | X | X | X | - | - | - | SEP exposures measured | - | X | Serious life events | Education Work status |
| *Idaiani (2021) (6)* | X | - | - | - | - | - | SEP exposures measured | - | X | - | Education  Income  Work status |
| *Kim (2020) (7)* | X | X | X | - | - | - | SEP exposures measured | - | X | Pollution | Income  Ownership  Subjective economic status |
| *Kim (2020) (C) (8)* | X | - | - | - | X | - | SEP exposures measured | - | - | Early life stress composite measure, early life stress (categorical), cortisol/saliva variables | Composite |
| *Leggett (2012) (9)* | X | X | X | X | - | - | SEP exposures measured | - | X (model 2 only) | Emotional support (model 2 only) Activities of daily living assistance (model 2 only) | Composite Education |
| **Paper** | **Sex** | **Age** | **Marital Status** | **Urban/rural or geographic area** | **Smoking** | **Ethnicity** | **Other SEP** | **Mental illness** | **Physical Health** | **Other** | **SEP Measures** |
| *Madyaningrum (2019) (10)* | X | X | X | X | - | - | Each adjusted for SEP exposures measured | - | X | Religion, physical exercise, number of social activities, sleep disturbances, sleep impairment, life satisfaction | Composite  Education |
| *Manaf (2016) (11)* | - | X | X | X | - | - | SEP exposures measured | - | - | - | Income  Work status |
| *Mubasyiroh (2022) (12)* | X | X | X | - | - | - | SEP exposures measured | X | X | Exposure to COVID-19 | Education Occupation Ownership |
| *Peltzer (2022) (C) (13)* | X | - | X | - | X | - | SEP exposures measured | - | - | Alcohol Physical activity Religion | Education  Income  Work status |
| *Sharma (2021) (14)* | X | X | X | - | - | X | SEP exposures measured | - | - | Corruption Index | Composite  Education  Financial difficulty |
| *Van (2021) (15)* | X | - | - | - | - | - | SEP exposures measured | - | - | Participating in social activities, family members, visit doctor in last 12 months, social supports score, barriers to access psychological services | Education Work status |
| *Vanoh (2016) (16)* | X | X | - | - | - | - | Income | - | - | Alcohol Living arrangements | Education |
| *Wichaidit (2022) (17)* | X | X | X | - | - | - | Education Income | - | - | - | Financial difficulty |
| *Yamada (2019) (18)* | X | X | X | X | - | X | SEP exposures measured | - | X | Living arrangement Number of household members | Composite  Education  Financial difficulty  Ownership  Work status |
| *Yeoh (2017) (19)* | - | X | X | - | - | - | Each occupation added separately into model | - | - | Stress, health locus of control | Occupation |

###

### Education

There were 21 papers that explored a measure of education in association with depression(1, 3-6, 9, 10, 12-16, 18, 20-26). One of these papers explored parental education, the remaining analysed personal education. Stratified results from Sasaki *et al* were also included here, despite the paper being rated as higher quality as these results only had adjusted estimates(27). Results in Figure 1 and Table 2 suggest that there was little evidence of an association between education and depression. Four papers provide statistical evidence that those with lower education levels have higher odds of depression(1, 6, 15, 24). Mubasyiroh *et al* (Table 2) found that having either senior or less than junior high school is protective of depression compared to having a diploma or university education(12).

The paper exploring parental education, from Indonesia, found that father’s education had no impact on their child’s chance of depression when assessed between 22 and 26 years of age (β=0.000, SE= 0.053, p=0.968) and although mother’s education showed a slightly negative association, there was limited statistical evidence to support this (β=-0.033, SE= 0.065, p=0.352)(2). However, the authors performed an additional analysis to determine if parental education had an influence dependent on the sex of the child. For both mothers and fathers, there was a positive association with male children (Mother: β= 0.096, SE= 0.086, p=0.050; Father: β= 0.053, SE= 0.074, p<0.281), which was in contrast to female children where the reverse association was demonstrated (Mother: β= -0.163, SE= 0.097, p=0.001; Father: β= -0.203, SE= 0.076, p=<0.001).

Figure 1. Associations between education and depression in papers that presented findings as odds ratio (lower quality papers)
†Papers are cross sectional unless stated. CC = case-control, C = cohort. Unadjusted ratios presented unless asterisked. *=adjusted association

Table 2. Associations between education and depression in papers with other estimates (lower quality papers)CS = cross sectional, β = standardised coefficient, B = unstandardised coefficient, U = unclear if standardised or unstandardised coefficient, CES-D = Centre for Epidemiological Studies Depression Scale

| **Main author (year)** | **Categories** | **Estimate** | **Results** | **Country** | **Sample Size** |
| --- | --- | --- | --- | --- | --- |
| Sharma (2021) (CS) (14) | Not completed high school (CES-D)  Not completed high school (Severe depression) | Coefficient (β)  (Adjusted) | -0.192 (0.217)  0.002 (0.018) | Vietnam | 1318 |
| Collier (2020) (CS) (25) | Years of education | Coefficient (β)  (Unadjusted) | -0.20 (p=<0.0001) | Vietnam | 977 |
| Van (2021) (CS) (15) | High school and over  Under high school | Coefficient (U)  (Adjusted) | Ref  1.41 (0.20, 2.62) | Vietnam | 376 |
| Mubasyiroh (2022) (CS) (12) | Diploma/University  Senior high  ≤Junior High | Coefficient (B)  (Adjusted) | Ref  -0.544 (-0.99, -0.10)  -1.563 (-2.68, -0.44) | Indonesia | 2743 |
| Duong (2020) (CS) (26) | Postgraduate  Elementary/Secondary | Coefficient (B)  (Adjusted) | Ref  2.39 (-1.26, 6.05) | Vietnam | 1385 |
| Leggett (2012) (CS) (9) | Education | Coefficient (β)  (Adjusted) | -0.16 (0.40) | Vietnam | 597 |
| Giang (2019) (CS) (4) | No schooling urban  Primary and above urban  No schooling rural  Primary and above rural | Probit  (Unclear) | Ref  -0.26 (-0.67, 0.14)  Ref  0.191 (-0.15, 0.54) | Vietnam | 2469 |
| Foong (2021) (CS) (3) | Primary and above among hard core poor  No formal education among hard core poor  Primary and above non-hard core poor  No formal education among non-hard core poor  Primary and above among Malay  No formal education among Malay  Primary and above among non-Malay  No formal education among -non Malay | PR  (Adjusted) | Ref  1.64 (1.23-2.19)  Ref  2.15 (1.57-2.93)  Ref  2.27 (1.66-3.10)  Ref  1.51 (1.11-2.06) | Malaysia | 2196 |

###

### Employment

#### Working status

Fourteen papers rated lower quality, including one cohort paper, explored working status in association with depression(1, 3-6, 11, 13, 15, 18, 22, 24-26, 28). From the results depicted in Figure 2 and Table 3, there was mixed evidence regarding the direction of association between working status and depression. Whilst some papers suggested that being unemployed increases the odds of depression, others suggested the reverse. Five estimates were supported by statistical evidence.

Figure 2. Associations between working status and depression in papers that presented findings as odds ratio (lower quality papers) †Papers are cross sectional unless stated. CC = case-control, C = cohort. Unadjusted ratios presented unless asterisked. *=adjusted association

Table 3. Associations between working status and depression in papers with other estimates (lower quality papers)
CS = cross sectional, PR = prevalence ratio, β = standardised coefficient, B = unstandardised coefficient

| **Main author (year)** | **Categories** | **Estimate** | **Results** | **Country** | **Sample Size** |
| --- | --- | --- | --- | --- | --- |
| Collier (2020) (CS) (25) | Employment status | Coefficient (β) (Unadjusted) | 0.21 (p<0.0001) | Vietnam | 977 |
| Duong (2020) (CS) (26) | Employed  Working from home  Student  Unemployed  Other | Coefficient (B)  (Unadjusted) | Ref  1.33 (0.35, 2.31)  2.11 (1.04, 3.18)  3.60 (2.06, 5.15)  2.24 (-0.86, 3.33) | Vietnam | 1385 |
| Manaf (2016) (CS) (11) | Employed  Unemployed | Coefficient (β)  (Adjusted) | Ref  -0.1 (-1.7, -0.2) | Malaysia | 340 |
| Giang (2019) (CS) (4) | Not currently working (Urban)  Currently working (urban)  Not currently working (rural)  Currently working (rural) | Probit  (Unclear) | Ref  0.16 (-0.14, 0.46)  Ref  -0.145 (-0.36, 0.07) | Vietnam | 2469 |
| Foong (2021) (CS) (3) | Currently working among hard core poor  Currently not working among hard core poor  Currently working among non-hard core poor  Currently not working among non-hard core poor  Currently working among malay  Currently not working among malay  Currently working among non-malay  Currently not working among -non malay | PR  (Adjusted) | Ref  1.00 (0.68, 1.47)  Ref  0.93 (0.68, 1.47)  Ref  0.99 (0.72, 1.37)  Ref  0.98 (0.68, 1.40) | Malaysia | 2196 |

#### Occupation

Three cross-sectional papers, and one case-control examined the relationship between occupation and depression(12, 19-21). Table 4 shows the results. In Yeoh *et al*, both professionals and housewives had a negative association with depression, with the former displaying statistical evidence of this association(19). Meanwhile, being a business owner had the opposite association. However, it is unclear which occupations were grouped under “Other occupations”, which should allow for cautious interpretations.

Table 4. Associations between occupation and depression (lower quality papers)
CS = cross sectional, C = cohort, cOR = crude odds ratio, B = unstandardised coefficient

| **Main author (year)** | **Categories** | **Estimate** | **Results** | **Country** | **Sample Size** |
| --- | --- | --- | --- | --- | --- |
| Razali (2022) (CS) (21) | Public sector (ref)  Private sector  Self-employed  Retired  Unemployed  Housekeeper  College/university students  Not working by choice  Other | cOR | 1.00  3.18 (2.18-4.66)  4.64 (2.94-7.36)  4.07 (2.25-7.49)  2.74 (0.88-8.87)  0.89 (0.37-2.01)  1.80 (1.16-2.77)  2.66 (0.56-13.65)  0.85 (0.35-1.91) | Malaysia | 963 |
| Mumang (2020) (CC) (20) | Private  Civil servant  Retired  Housewife  Unemployed | cOR | Ref  1.07 (0.47-2.24)  4.55 (1.10-26.60)  1.19 (0.68-2.10)  2.15 (0.71-6.91) | Indonesia | 320 |
| Mubasyiroh (2022) (CS) (12) | Civil servants BUMN /BUMD  Private/self employed  Informal sectors  Currently laid off/ job seekers  Unemployed  Students | Coefficient (B)  (Adjusted) | Ref  0.702 (0.288, 1.116)  0.523 (-0.290, 1.326)  1.831 (1.150, 2.512)  1.401 (0.883, 1.920)  1.258 (0.582, 1.934) | Indonesia | 2743 |
| Yeoh (2017) (CS) (19) | Other occupations  Housewife  Business Owner  Professional | Coefficient (B)  (Adjusted) | Ref  -1.314 (-4.64, 2.02)  4.170 (1.10, 7.24)  -5.603 (-8.82 ,-2.39) | Malaysia | 728 |

### Financial Status

#### Income

The association between income and depression was explored in 13 papers rated as lower quality, (1, 3, 6, 7, 11, 13, 20, 22-24, 26, 28, 29). Several different measures of income were used, and within all, mixed evidence was present; no single measure consistently suggested evidence of a statistical association with depression (Figures 3 and 4).

Kim *et al* stratified their analyses by overall depressive symptoms, clinical depression and mild depressive symptoms, and by sex, but differences in results were minimal, with just a small suggestion of a negative association between increased household per capita expenditure and decreased rates of mild depressive symptoms in total and in women(7). Idaiani *et al* also looked at expenditure and found that those who had the least expenditure, and were categorised as poorest, had increased chances of depression (6). However, similar to Manaf *et al*, it was not known which ages are included in these papers, and whether they fully meet the inclusion criteria for the review(11).

Foong *et al* presented their results as prevalence ratios (PR), and therefore are not shown in the figures(3). The PR were stratified by ethnicity (Malay or not Malay). Results suggest that hardcore poor individuals, i.e. those who did not reach the poverty line cut off (income less than MYR 460), had increased prevalence of depression, compared to those classed as non-poor in both Malays (PR: 1.34, 95% confidence interval (CI): 1.03-1.76) and non-Malays (PR: 1.68 95% CI: 1.30-2.19).

For Peltzer *et al*, the CI for the 45-59 age category longitudinal estimate did not include the odds ratio (OR) provided, and therefore could not be included in the forest plot (Figure 3)(13).

Figure 3. Associations between income and depression in papers that presented findings as odds ratio (lower quality papers)
†Papers are cross sectional unless stated. CC = case-control, C = cohort, RM = Ringgit Malaysia, NS = not specified.
Unadjusted ratios presented unless asterisked. *=adjusted association

Figure 4. Associations between income and depression in papers with beta coefficients (lower quality papers)
All papers are cross sectional.
Unadjusted ratios presented unless asterisked. *=adjusted association VND = Vietnamese dong.

#### Financial difficulty

Six papers explored the association between financial difficulties and depression, and were rated as lower quality(4, 14, 17, 18, 23, 25). Evidence from Collier *et al* suggested that suffering from serious financial problems was associated with an increase in depression symptoms (β= 0.39, p=<0.0001)(25).Similarly, experiencing food insecurity was a risk factor for increased odds of this outcome (Figure 5). Associations with economic dependence appears to depend on the cut off the authors used, and may only have an impact on more severe depression i.e a higher cut off; Yamada *et al* found that at the highest cut off, being partially or fully dependent on others financially resulted in lower odds of depression, with statistical evidence to support this(18). Wichaidit *et al* was conducted during the COVID-19 pandemic and found that, although individuals who experienced economic distress had higher odds of depression, there was limited statistical evidence to support this, regardless of whether they had access to emergency cash reserves or not(17). However, this paper did not use a valid and reliable measure to assess depression since they utilised a tool that consisted of only two questions. Individuals from poor households had poorer mental health, when using both the CES-D index continuously (β= 0.82, SE: 0.373), and using a cut off for severe depression (β= 0.07, SE: 0.036)(14).

Results from a probit model, using data from Vietnam, suggested that having enough finance for daily living was indicative of a lower chance of depression in urban and rural areas (Urban -0.440, 95% CI: -0.726, -0.154; Rural -0.382, 95% CI: -0.643, -0.123) as was receiving monetary support from children, but this was not supported by statistical evidence (Urban -0.155, 95% CI: -0.523, 0.212; Rural -0.041, 95% CI: -0.233, 0.151)(4). These results were not compared to the highest reference category as the probit results could not be inverted.

Figure 5. Associations between financial difficulty and depression in papers that presented findings as odds ratios (lower quality papers).
All papers are cross sectional.
Unadjusted ratios presented unless asterisked. *=adjusted association

#### Subjective Economic Status

Kim *et al* demonstrated similar results to the high quality papers, although magnitude of associations were less pronounced for the clinical depression outcome, and there was no association between income ladder and mild depression in males(7).

Figure 6. Associations between subjective economic status and depression (lower quality papers)
†All papers are cross sectional.
*=adjusted association

### Asset based measures

#### Composite

Five papers explored the relationship between a composite measure of SEP and depression. Table 5 details what was used in each composite measure(8-10, 14, 18).

Table 5. Composite measures

| **Main author (year)** | **Measure** | **Components** |
| --- | --- | --- |
| Sharma (2021) (14) | Number of Assets | Ownership of:  Bicycle, black and white TV, colour TV, scooter/motorcycle/moped, landline telephone, mobile phone, electric fan, radio/stereo, pump set, refrigerator, computer/laptop, internet access, washing machine, cooler/air conditioner, car/truck/van, and flush toilet |
| Kim (2020) (8) | Multidimensional summary measure | Combination of household income (weekly income in pesos standardized to year), a household assets (sum of the following items: home ownership, electricity in the home, type of housing material, and ownership of items such as air conditioner, television, refrigerator, or car), and educational attainment at both years (years of formal schooling); for socioeconomic status at age 21, own education was used, not parents. |
| Yamada (2019) (18) | Wealth Index | Ownership of:  Bicycle, radio, store-bought furniture, electric fan, two wheelers, four wheelers, color television, video/DVD player, mobile phone, refrigerator, rice cooker, motorized pump, washing machine, and computer/laptop |
| Madyaningrum (2019) (10) | Socioeconomic status | Total value of household assets (no further detail provided) |
| Leggett (2012) (9) | Material Hardship | 8 items including lack of access to water and food, and whether individual had ownership of consumer items e.g TV or bicycle |

A study conducted in the Philippines using data from the Cebu Longitudinal Health and Nutrition Survey explored how early life (up to age 11), and current SEP (at age 21) was associated with depression at age 21(8). The authors found that SEP in the early life (β=-0.049, SE=0.49, p=>0.05) and at age 21 was negatively associated with risk of depression, but it was only the latter that had statistical evidence of an association with a coefficient of -0.64 and a SE of 0.20 (p=<0.02), after adjusting for confounders.

The paper from Vietnam suggests that the ownership of more household asset/access to assets was associated with a decreased chance of depression (β = 0.28, SE: 0.21)(9). However, in the results in Sharma *et al,* the negative association was very small, when using the CES-D score and a measurement of severe depression (CES-D - β:-0.090, SE: 0.056; Severe depression – β=-0.007, SE: 0.005)(14). A paper from Indonesia that uses socioeconomic status as an aggregate measure supported this finding, with those in the lowest category of household asset ownership having double the odds of depression (adjusted OR (aOR): 2.04, 95% CI: 1.49-2.78)(10); this paper was in the elderly population. These results were replicated further in a study of older adults in Myanmar(18). Regardless of the categorisation of depression, there was statistical evidence for a higher wealth score resulting in reduced odds of depression (0/1 aOR: 0.85, 95% CI: 0.77-0.93; 1/2 aOR: 0.83, 95% CI: 0.75-0.92; 2/3 aOR: 0.84, 95% CI: 0.73-0.95).

#### Ownership

Three papers rated lower quality used ownership as an indicator of SEP(7, 12, 18). Results suggested that having an outside kitchen was not associated with depression, but having outside water was associated with a reduced chance of depression, when defining depression as overall depressive symptoms or mild depressive symptoms (Figure 7). Outside kitchen or water was not associated with clinical depressive symptoms. As it was unclear whether results from Kim *et al* were standardised or unstandardised, the results for Mubasyiroh *et al*, which are unstandardised, are presented separately. They found that having home ownership categorised as rented was associated with increased depression, compared to not rented, after adjustment for confounders (B= 0.783, 95% CI: 0.321, 1.204)(12).

Yamada *et al* analysed land ownership and used different cut off points for experiencing depression(18). At the higher cut off points (either 2 or 3), not owning land was associated with higher odds of depression, with statistical evidence to support the association. The aOR and CI, for each cut off (0/1, 1/2, 2/3) respectively were: 1.50 (0.68-3.32), 2.60 (1.67-4.04) and 2.25 (1.74-2.91).

Figure 7. Associations between ownership and depression (lower quality papers)
All papers are cross sectional.
*=adjusted association

## Associations of SEP with Suicidal Ideation

There were three papers on suicidal ideation with one each from Malaysia, Thailand and the Philippines(30-32). All three papers used a cross sectional study design and were rated as lower quality. Two papers recruited participants from the general population, whilst the other recruited from a healthcare population. The Filipino paper analysed birth cohort data cross-sectionally and explored suicidal ideation in those around 18 year old(31). The other two papers investigated the associations in a more general adult population. Two papers did not provide crude estimate and Table 6 highlights what their estimates were adjusted for.

Table 6. Variables that have been controlled for in papers that only have adjusted estimates

| **Main author (year)** | **Sex** | **Age** | **Marital Status** | **Urban/rural or geographic area** | **Smoking** | **Ethnicity** | **Other SEP** | **Mental illness** | **Physical Health** | **Other** | **SEP Measures** |
| --- | --- | --- | --- | --- | --- | --- | --- | --- | --- | --- | --- |
| *Peltzer (2022) (30)* | X | X | X | - | - | - | Each adjusted for SEP exposures measured | X | X | Social support  Care setting | Education  Financial difficulty  Work status |
| *Cheung (2009) (31)* | X | X | - | - | - | - | Mothers education Wealth index Housing materials | - | X | Breastfeeding, witness domestic violence, Length Z-score at birth, postnatal length gain in Z-score | Education |

### Education

All three papers on suicidal ideation suggested associations between lower levels of education and increased odds of suicidal ideation (Figure 8) (30-32). There was statistical evidence of this association in Cheung *et al,* but CI are wide(31). There was weak statistical evidence of the association in the paper from Thailand(30).

Cheah *et al* also presented results stratified by sex and age (Figure 9)(32). When stratifying by sex, both males and females with primary education had increased odds of suicidal ideation, compared to those with higher levels, although neither provided statistical evidence of an association. Similar results were demonstrated with age, although in the age 18-31 category, results suggested that primary education resulted in reduced odds of suicidal ideation. Statistical evidence of this association was present for the age 31-40 and 51 and above age categories.

Figure 8. Association between education and suicidal ideation.
†All papers are cross sectional. Unadjusted associations shown unless asterisked. *=adjusted association

###

Figure 9. Association between education stratifications and suicidal ideation
Unadjusted associations shown. All papers are cross sectional

### Financial Status

#### Income

One paper from Malaysia assessed income in association with suicidal ideation(32). Cheah et al*,* as above, stratified their findings by income. The direction of association differs between males and females (Figure 10). For males, lower income was associated with increased odds of suicidal ideation, which was consistent with the total sample, while for females the associated is reversed. However, for both samples, CI cross zero suggesting limited statistical evidence of an association in either direction. When stratified by age category (18-30, 31-40, 41-50, ≥51), the association was reversed in each age group i.e lower incomes had reduced odds of suicidal ideation. However, as with the whole sample, CI were wide and there was limited statistical evidence that income was associated with suicidal ideation, regardless of age category.

####

Figure 10. Association between income and suicidal ideation
Unadjusted associations shown. All papers are cross sectional

#### Financial Difficulty

A single paper explored debt status and its’ association with suicidal ideation in those who attended a primary care or religious centre (30). In this paper, there was limited statistical evidence to suggest a difference between odds of suicidal ideation between those with little/no self-reported debt, and those with a higher debt status (aOR: 1.21, CI: 0.74-1.99.)

### Employment

#### Working Status

Associations with employment were looked at the paper conducted in Thailand(30). The paper found that not being employed increased the odds of suicidal ideation by 92% (aOR: 1.92, CI: 1.18-3.13.

## References

1. Cheah Y, Azahadi M, Phang S, Abd Manaf N. SOCIODEMOGRAPHIC, LIFESTYLE AND HEALTH FACTORS ASSOCIATED WITH DEPRESSION AMONG ADULTS IN MALAYSIA: AN ETHNIC COMPARISON. Journal of Health and Translational Medicine. 2019;22(1).

2. Fakhrunnisak D, Patria B. The positive effects of parents’ education level on children’s mental health in Indonesia: a result of longitudinal survey. BMC Public Health. 2022;22(1):949.

3. Foong HF, Hamid TA, Ibrahim R, Haron SA. The intersectional effects of ethnicity/race and poverty on health among community-dwelling older adults within multi-ethnic Asian populace: a population-based study. BMC Geriatrics. 2021;21(1):516.

4. Giang LT, Nguyen TT, Tran NTT. Factors Associated with Depression among Older People in Vietnam. Journal of Population and Social Studies [JPSS]. 2019;27(2):181 - 94.

5. Haseen F, Prasartkul P. Predictors of depression among older people living in rural areas of Thailand. Bangladesh Medical Research Council Bulletin. 2011;37(2):51-6.

6. Idaiani S, Indrawati L. Functional status in relation to depression among elderly individuals in Indonesia: a cross-sectional analysis of the Indonesian National Health Survey 2018 among elderly individuals. BMC Public Health. 2021;21(1):2332.

7. Kim Y, Manley J, Radoias V. Air Pollution and Long Term Mental Health. Atmosphere [Internet]. 2020; 11(12).

8. Kim AW, Adam EK, Bechayda SA, Kuzawa CW. Early life stress and HPA axis function independently predict adult depressive symptoms in metropolitan Cebu, Philippines. American Journal of Physical Anthropology. 2020;173(3):448-62.

9. Leggett A, Zarit SH, Nguyen NH, Hoang CN, Nguyen HT. The influence of social factors and health on depressive symptoms and worry: A study of older Vietnamese adults. Aging & Mental Health. 2012;16(6):780-6.

10. Madyaningrum E, Chuang Y-C, Chuang K-Y. Prevalence and Related Factors of Depression among the Elderly in Indonesia. International Journal of Gerontology. 2019;13(3):202-6.

11. Manaf M, Qureshi A, Loftizadeh M, Ganaegeran K, Yadav H, Al-Dubai S. Factors associated with anxiety and depression among outpatients in Malaysia: a cross-sectional study. Malaysian Journal of Public Health Medicine. 2016;16(3):181-7.

12. Mubasyiroh R, Suryaputri IY, Idaiani S, et al. Mental Health Disorders of the Indonesian People in the Early Stages of the COVID-19 Pandemic: Who is Vulnerable to Experiencing it? International Journal of Mental Health Promotion. 2022;24(5).

13. Peltzer K, Pengpid S. Socioeconomic position and physical and mental health among middle-aged and older adults: Cross-sectional and longitudinal results from a national community sample in Thailand. Journal of Human Behavior in the Social Environment. 2022:1-15.

14. Sharma S, Singhal S, Tarp F. Corruption and mental health: Evidence from Vietnam. Journal of Economic Behavior & Organization. 2021;185:125-37.

15. Van NHN, Huyen NTK, Luong NT, Duc DM, Thanh PQ. Factors associated with depression among the elderly living in rural Vietnam 2019: Recommendations to remove barriers of psychological service accessibility. International Journal of Mental Health. 2021;50(2):136-50.

16. Vanoh D, Shahar S, Yahya HM, Hamid TA. Prevalence and Determinants of Depressive Disorders among Community-dwelling Older Adults: Findings from the Towards Useful Aging Study. International Journal of Gerontology. 2016;10(2):81-5.

17. Wichaidit W, Prommanee C, Choocham S, Chotipanvithayakul R, Assanangkornchai S. Modification of the association between experience of economic distress during the COVID-19 pandemic and behavioral health outcomes by availability of emergency cash reserves: findings from a nationally-representative survey in Thailand. PeerJ. 2022;10:e13307.

18. Yamada H, Yoshikawa K, Matsushima M. Geriatric Depressive Symptoms in Myanmar: Incidence and Associated Factors. Journal of Applied Gerontology. 2019;39(11):1230-9.

19. Yeoh SH, Tam CL, Wong CP, Bonn G. Examining Depressive Symptoms and Their Predictors in Malaysia: Stress, Locus of Control, and Occupation. Frontiers in Psychology. 2017;8.

20. Mumang AA, Liaury K, Syamsuddin S, et al. Socio-economic-demographic determinants of depression in Indonesia: A hospital-based study. PLOS ONE. 2020;15(12):e0244108.

21. Razali S, Tukhvatullina D, Hashim NA, et al. Sociodemographic Factors of Depression During the COVID-19 Pandemic in Malaysia: the COVID-19 Mental Health International Study. East Asian Arch Psychiatry. 2022;32(4):82-8.

22. Imran A, Azidah AK, Asrenee AR, Rosediani M. Prevalence of depression and its associated factors among elderly patients in outpatient clinic of Universiti Sains Malaysia Hospital. Med J Malaysia. 2009;64(2):134-9.

23. Mesbah SF, Sulaiman N, Mohd Shariff Z, Ibrahim Z. Does Food Insecurity Contribute towards Depression? A Cross-Sectional Study among the Urban Elderly in Malaysia. International Journal of Environmental Research and Public Health [Internet]. 2020; 17(9).

24. Sidik S, Zulkefli N, Shah SA. Factors associated with depression among elderly patients in a primary health care clinic in Malaysia. Asia Pacific Family Medicine. 2003;2(3):148-52.

25. Collier KM, Weiss B, Pollack A, Lam T. Explanatory variables for women’s increased risk for mental health problems in Vietnam. Social Psychiatry and Psychiatric Epidemiology. 2020;55(3):359-69.

26. Duong K, Bao T, Nguyen P, et al. Psychological Impacts of COVID-19 During the First Nationwide Lockdown in Vietnam: Web-Based, Cross-Sectional Survey Study. JMIR Form Res. 2020;4(12):e24776.

27. Sasaki Y, Shobugawa Y, Nozaki I, et al. Association between depressive symptoms and objective/subjective socioeconomic status among older adults of two regions in Myanmar. PLOS ONE. 2021;16(1):e0245489.

28. Sherina MS, Rampal L, Mustaqim A. The prevalence of depression among the elderly in Sepang, Selangor. Med J Malaysia. 2004;59(1):45-9.

29. Prueksaritanond S, Kongsakol R. Biopsychosocial impacts on the elderly from a tsunami-affected community in southern Thailand. J Med Assoc Thai. 2007;90(8):1501-5.

30. Peltzer K, Pengpid S. Suicidal ideation and associated factors among clients of primary care and religious care centers in Thailand. Asian Journal of Social Health and Behavior. 2022;5(2):57-62.

31. Cheung YB, Ashorn P. Linear growth in early life is associated with suicidal ideation in 18-year-old Filipinos. Paediatric and Perinatal Epidemiology. 2009;23(5):463-71.

32. Cheah YK, Azahadi M, Phang SN, Abd Manaf NH. Association of Suicidal Ideation with Demographic, Lifestyle and Health Factors in Malaysians. East Asian Arch Psychiatry. 2018;28(3):85-94.
