# Supplementary material for: The association between socioeconomic position and depression or suicidal ideation in low- and middle-income countries in Southeast Asia: a systematic review and meta-analysis
Source: BMC Public Health. 2024 Dec 18;24:3507. doi: 10.1186/s12889-024-20986-9 (PMC11656959; doi:10.1186/s12889-024-20986-9)
Supplement: Supplementary file 4 — Supplementary Material 4. [file 12889_2024_20986_MOESM4_ESM.docx]

# Additional files 4

## Publication bias

Visual inspection of the plots indicated that studies included in the meta-analyses may have tended to be larger than those not included, although Egger’s test provided limited evidence of asymmetry for either plot (education: p=0.334, working status: p=0.388).

Figure 1. Funnel plot assessing small-study effects in estimates from higher rated papers included in the education meta-analysis

Figure 2. Funnel plot assessing small-study effects in estimates from higher rated papers included in the working status meta-analysis

## Sensitivity Analysis

The meta-analysis was repeated for education and working status, including the papers rated as lower quality. Results for education remained consistent when just including the higher quality papers (Figure 1). For working status, whilst the direction of overall association remained the same, including the papers rated as lower quality results in greater statistical evidence of the association (Figure 2).

Figure 3. Meta-analysis of the association between education and depression, including all papers, in papers that presented findings as odds ratios. † Papers are cross sectional unless stated. CC = case control. C= cohort.
Unadjusted ratios presented unless asterisked. *=adjusted association

Figure 4. Meta-analysis of the association between working status and depression, including all papers, in papers that presented findings as odds ratios †Papers are cross sectional unless stated. CC = case control. C= cohort.
Unadjusted ratios presented unless asterisked. *=adjusted association
