# Supplementary material for: The association between socioeconomic position and depression or suicidal ideation in low- and middle-income countries in Southeast Asia: a systematic review and meta-analysis
Source: BMC Public Health. 2024 Dec 18;24:3507. doi: 10.1186/s12889-024-20986-9 (PMC11656959; doi:10.1186/s12889-024-20986-9)
Supplement: Supplementary file 5 — Supplementary Material 5. [file 12889_2024_20986_MOESM5_ESM.docx]

Table 1. Study characteristics
NS = not stated, SD = standard deviation, IQR = interquartile range, DASS = Depression, Anxiety and Stress Scale, M-GDS = Malay Geriatric Depression Scale , TGDS = Thai Geriatric Depression Scale , GDS = Geriatric Depression Scale, PHQ= Patient Health Questionnaire, CES-D= Centre for Epidemiological Studies Depression Scale, MINI = Mini-International Neuropsychiatric Interview , DSM-IV-TR = Diagnostic and Statistical Manual of Mental Disorders, HRSD = Hamilton Rating Scale for Depression, HADS-D = Hospital Anxiety and Depression Scale, SDS = Self-rating Depression Scale , BDI = Beck Depression Inventory

| **Main authors (year)** | **Country** | **Sampling frame** | **Male %** | **Sample size (Response %)** | **Outcome** | **Tool used (Outcome definition)** | **Age (Mean (SD), median (IQR), or %)** | **Measures of SEP** | **Quality Rating** |
| --- | --- | --- | --- | --- | --- | --- | --- | --- | --- |
| **Cross-sectional** | | | | | | | |  |  |
| *Idaiani (2021) (1)* | Indonesia | Population | NS | 93,829 (93.2) | Depression | MINI v6 (at least 2 “yes” from the first 3 questions plus 2 “yes” to questions 4 to 10) | Mean: 68.5 (NS) | Education  Income  Work status | Lower |
| *Kim (2020) (2)* | Indonesia | Population | 42.1 | 7,969 (NS) | Depression | CES-D 10 (≥10, continuous) | NS | Income  Ownership Subjective economic status | Lower |
| *Madyaningrum (2019) (3)* | Indonesia | Population | 48.4 | 2,910 (NS) | Depression | CES-D 10 (≥10) | Mean: 67.4 (6.1) | Composite  Education | Lower |
| *Mahwati (2017) (4)* | Indonesia | Population | 46.0 | 3,103 (NS) | Depression | CES-D 10 (>10) | Categorical: 60-69: 63.7%  ≥70: 36.3% | Education Income  Work status | Higher |
| *Mardiana (2022) (5)* | Indonesia | Population | 56.9 | 70,090 (NS) | Depression | MINI (2 “Yes” for the questions 1-3 + 2 Yes for questions 4-10) | Mean: 51.5 (4.3) | Education  Income Ownership  Work status | Higher |
| *Mubasyiroh (2022) (6)* | Indonesia | Population | 30.8 | 2,743 (NS) | Depression | PHQ-9 (≥10) | Categorical: 18:24: 23.8%  25-34: 36.1%  35-44: 21.3%  45-54: 13.8%  ≥55: 5.1% | Education Occupation Ownership | Lower |
| *Abdul Manaf (2016) (7)* | Malaysia | Population | 40.0 | 230 | Depression | DASS-21 (≥5) | Mean: 69.1 (7.3) | Education  Income  Work status | Higher |
| *Ahmad (2020) (8)* | Malaysia | Population | 47.1 | 3,772 (94.8) | Depression | M-GDS-14 (≥6) | Mean: 68.3 (6.9) | Education  Income | Higher |
| *Cheah (2018) (9)* | Malaysia | Population | 46.1 | 10,141 (93.0) | Suicidal Ideation | In the past 30 days, have you thought about suicide? (Yes) | Mean: 41.2 (15.4) | Education  Income | Lower |
| *Cheah (2019) (10)* | Malaysia | Population | 46.1 | 10,141 (NS) | Depression | Were you ever depressed or down, most of the time, nearly every day, for 2 weeks? (Yes) | Mean: 41.2 (15.4) | Education  Income  Work status | Lower |
| *Foong (2021) (11)* | Malaysia | Population | 47.0 | 2,196 (90.0) | Depression | GDS-15 (≥4) | Categorical: 60-70: 62.8%  ≥71: 37.2% | Education Income  Work status | Lower |
| *Imran (2009) (12)* | Malaysia | Healthcare | 32.8 | 244 (NS) | Depression | M-GDS-14 (≥8) | Categorical: 60-64: 43.9%  65-69: 31.6%  70-74: 13.1%  75-79: 8.2%  ≥80: 3.2% | Education  Income  Work status | Lower |
| *Maideen (2014) (13)* | Malaysia | Population | 37.2 | 1,460 (61.9) | Depression | PHQ-9 (Malay) (≥10) | Mean: 35.4 (13.8) | Education  Financial difficulty  Work status | Higher |
| *Manaf (2016) (14)* | Malaysia | Healthcare | 62.1 | 340 (NS) | Depression | HADS-D (Continuous) | Categorical: <30: 41.5%  30-49: 32.6%  ≥50: 25.9% | Income  Work status | Lower |
| *Md Aris (2014) (15)* | Malaysia | Healthcare | 54.2 | 452 (NS) | Depression | PHQ-9 (Malay) (≥10) | Median: 33.0 (19.0) | Education  Occupation | Higher |
| *Mesbah (2020) (16)* | Malaysia | Population | 42.3 | 220 (NS) | Depression | GDS-15 (≥5) | Median: 65.5 (8.0) | Education Financial difficulty  Income | Lower |
| *Rashid (2015) (17)* | Malaysia | Population | 32.0 | 2,005 (NS) | Depression | GDS-30 (>19) | Categorical: 60-69: 62.3%  70-79: 30.5%  ≥80: 7.2% | Education  Work status | Higher |
| *Razali (2022) (18)* | Malaysia | Population | 32.3 | 963 (NS) | Depression | CES-D (>23/24 + algorithm score of ≥9.03) | Mean: 40.1 (NS) | Education  Occupation | Lower |
| *Said (2022) (19)* | Malaysia | Population | 46.5 | 6,551 (NS) | Depression | DASS-21 (NR) | Mean: 65.5 (7.7) | Education  Income  Work status | Higher |
| *Sherina (2004) (20)* | Malaysia | Population | 44.8 | 223 (84.8) | Depression | GDS-30 (Malaysia) (>10) | Mean: 69.7 (6.8) | Income  Work status | Lower |
| *Sidik (2003) (21)* | Malaysia | Healthcare | NA | 845 (NS) | Depression | PHQ-9 (≥ 10) | Mean: 30.9 (10.4) | Education  Income  Work status | Lower |
| *Sidik (2004) (22)* | Malaysia | Population | 44.8 | 223 (84.8) | Depression | GDS-30 (>10) | Mean: 69.7 (6.8) | Education  Income  Work status | Higher |
| *Sidik (2012) (23)* | Malaysia | Healthcare | 56.2 | 208 (87.5) | Depression | GDS 30 (>10) | NS | Education  Financial difficulty  Work status | Higher |
| *Sok Yee (2011) (24)* | Malaysia | Population | 43.8 | 520 (NS) | Depression | Malay HADS-D (≥8) | Mean: 41.9 (17.6) | Education  Income  Work status | Higher |
| *Ting (2014) (25)* | Malaysia | Population | 40.8 | 223 (NS) | Depression | PHQ-9 (Malay) (≥10) | Mean: 46.7 (15.5) | Education Income  Work status | Higher |
| *Vanoh (2016) (26)* | Malaysia | Population | 48.0 | 2,264 (NS) | Depression | GDS-15 (≥5) | Mean: Case: 69.8 (6.4)  Control: 68.9 (6.2) | Education | Lower |
| *Yeoh (2017) (27)* | Malaysia | Population | 37.5 | 728 (NS) | Depression | BDI-II (Continuous) | Mean: 30.8 (13.2) | Occupation | Lower |
| *Cho (2021) (28)* | Myanmar | Population | 33.7 | 655 (NS) | Depression | GDS-15  (>4) | Mean: 70.2 (NS) | Education  Work status | Higher |
| *Sasaki (2021) (29)* | Myanmar | Population | 40.5 | 1,186 (Yangon: 98.4, Bago: 86.5) | Depression | GDS-15 (≥ 5) | Categorical:60-69: 56.0%  70-79: 31.7%  ≥80: 12.6% | Composite  Education Subjective economic status | Higher |
| *Yamada (2019) (30)* | Myanmar | Population | 43.2 | 998 (NS) | Depression | GDS-4 (0/1, 1/2 or 2/3) | Mean: 69.7 (8.3) | Composite  Education  Financial difficulty Ownership  Work status | Lower |
| *Cheung (2009) (31)* | Philippines | Population | 53.1 | 1,536 (NS) | Suicidal Ideation | ‘You felt life isn't worth living’, ‘You wished you were dead’ and ‘You had the idea of taking your own life’ (Most of the time to at least one statement) | Mean: 18.2 (0.4) | Education | Lower |
| *Anantapong (2016) (32)* | Thailand | Healthcare | 29.1 | 604 (NS) | Depression | TGDS-15 (≥6) | Mean: 72.3 (5.5) | Education | Higher |
| *Aung (2016) (33)* | Thailand | Population | 45.1 | 435 (97.7) | Depression | TGDS-30 (Continuous) | Mean: 83.8 (3.5) | Education | Higher |
| *Charoensakulchai (2019) (34)* | Thailand | Population | 45.9 | 416 (NS) | Depression | TGDS-30 (>12) | Mean: 69.3 (7.0) | Education  Financial difficulty | Higher |
| *Haseen (2011) (35)* | Thailand | Population | 44.5 | 1,001 (NS) | Depression | EURO-D (5/6) | Categorical: 60-69: 54.5%  70-79: 35.8%  ≥80: 9.8% | Education Work status | Lower |
| *Naviganuntana (2022) (36)* | Thailand | Population | 29.3 | 888 (91.3) | Depression | TGDS (≥13) | Categorical: 60-69: 53.3%  70-79: 32.8%  ≥80: 13.9% | Education Income  Work status | Higher |
| *Peltzer (2022) (37)* | Thailand | Healthcare | 25.3 | 1,214 (NS) | Suicidal Ideation | How often over the last 2 weeks they have been bothered by thoughts that they would be better off dead or hurting oneself in anyway? (Several days, more than half the days or nearly every day) | Categorical: 19-44: 42.1%  45-59: 45.3%  60-93: 12.6% | Education  Financial difficulty  Work status | Lower |
| *Prueksaritanond (2007) (38)* | Thailand | Population | 35.6 | 38 (NS) | Depression | Zung SDS (≥50) | Mean: 67.6 (6.5) | Income | Lower |
| *Wichaidit (2022) (39)* | Thailand | Population | 48.3 | 1,555 (68.3) | Depression | PHQ-2 (≥3) | Mean: 41.3 (0.3) | Financial difficulty | Lower |
| *Collier (2020) (40)* | Vietnam | Population | 43.1 | 977 (97.7) | Depression | PHQ-9 (Continuous) | Median: 42.0 (NS) | Education  Financial difficulty  Work status | Lower |
| *Dao (2018) (41)* | Vietnam | Population | 48.8 | 299 (NS) | Depression | Zung SDS (≥50) | Mean: 70.6 (7.4) | Education  Occupation | Higher |
| *Do (2022) (42)* | Vietnam | Population | 31.3 | 495 (NS) | Depression | GDS-15 (≥5) | Categorical: 60-69: 52.5%  70-79: 35.6%  ≥80: 11.9% | Education | Higher |
| *Duong (2020) (43)* | Vietnam | Population | 63.5 | 1,385 (NS) | Depression | DASS-21 (>9) | Median: 28.0 (NS) | Education Income  Work status | Lower |
| *Giang (2019) (44)* | Vietnam | Population | 40.2 | 2,469 (96.3) | Depression | How often do you feel sad or depressed in later life? (Sometimes or Most of time) | Range: 60-108 | Education Financial difficulty  Work status | Lower |
| *Hoang (2022) (45)* | Vietnam | Population | 100.0 | 1,080 (97.7) | Depression | DASS-21 (Continuous) | Mean: 35.5 (14.6) | Education Financial difficulty  Occupation | Higher |
| *Leggett (2012) (46)* | Vietnam | Population | 50.0 | 597 (NS) | Depression | CES-D 20 (Continuous) | Mean: 70.3 (9.1) | Composite Education | Lower |
| *Sharma (2021) (47)* | Vietnam | Population | 48.0 | 1,318 (NS) | Depression | CES-D 10 (≥10) | Mean: 38.7 (10.6) | Composite  Education Financial difficulty | Lower |
| *Tran (2022) (48)* | Vietnam | Population | 42.5 | 4,962 (NS) | Depression | CES-D-11 (≥8.8) | Categorical: 60-69: 43.6%  70-79: 33.1%  ≥80: 23.3% | Education Income | Higher |
| *Van (2021) (49)* | Vietnam | Population | 33.5 | 376 (98.9) | Depression | PHQ-9 (≥10) | Categorical: 60-70: 47.3%  >70: 52.7% | Education Work status | Lower |
| *Vu (2019) (50)* | Vietnam | Population | 30.0 | 523 (NS) | Depression | GDS-4 (4) | Mean: 71.0 (8.2) | Financial difficulty  Occupation Ownership | Higher |
| *Yen Phi (2017) (51)* | Vietnam | Healthcare | 47.7 | 512 (NS) | Depression | DSM-5 (NA) | Mean: 46.4 (13.9) | Education Subjective economic status  Work status | Higher |
| **Cohort** | | | | | | | |  |  |
| *Fakhrunnisak (2022) (52)* | Indonesia | Population | 52.5 | 767 (NS) | Depression | CES-D 8 (Continuous) | Range: 22-26 | Parental education | Lower |
| *Isaura (2019) (53)* | Indonesia | Population | 53.6 | 8,613 (NS) | Depression | CES-D 10 (≥10) | Categorical: <40: 45.8%  40-59: 3.8%  50-59: 0.3%  ≥60: 0.07% | Financial difficulty | Higher |
| *Patria (2022) (54)* | Indonesia | Population | 46.7 | 18,374 (NS) | Depression | CES-D 10 (Continuous) | Mean: 34.6 (13.0) | Education | Higher |
| *Kim (2020) (55)* | Philippines | Population | 52.1 | 1,244 (NS) | Depression | CES-D 16 (Continuous) | Mean: 21.5 (0.3) | Composite | Lower |
| *Jittawisuthikul (2011) (56)* | Thailand | Population | 37.2 | 358 (99.6) | Depression | Thai EURO-D scale (5/6) | Mean: 69.6 (7.4) | Composite | Higher |
| *Peltzer (2022) (57)* | Thailand | Population | 47.8 | Cross-sectional = 5616 (72.3) Longitudinal = 3708 | Depression | CES-D-10 (≥10) | Categorical: 45-59: 31.8%  60-74: 38.9%  75-117: 9.2% | Education  Income Work status | Lower |
| **Case-Control** | | | | | | | |  |  |
| *Lueboonthavatchai (2009) (58)* | Thailand | Healthcare | 21.1 | 180 (NS) | Depression | DSM-IV-TR + Thai HRSD (Thai score 8 or more) | Mean: 42.8 (12) | Education Income Financial difficulty  Work status | Higher |
| *Mumang (2020) (59)* | Indonesia | Healthcare | 37.2 | 320 (NS) | Depression | Indonesian mental disorder guidelines for classification and diagnosis (3rd edition) (NA) | Categorical: <32: 24.3%  32-42: 27.4%  43-52: 24.1%  >52: 24.3% | Education  Income Occupation | Lower |

1. Idaiani S, Indrawati L. Functional status in relation to depression among elderly individuals in Indonesia: a cross-sectional analysis of the Indonesian National Health Survey 2018 among elderly individuals. BMC Public Health. 2021;21(1):2332.

2. Kim Y, Manley J, Radoias V. Air Pollution and Long Term Mental Health. Atmosphere [Internet]. 2020; 11(12).

3. Madyaningrum E, Chuang Y-C, Chuang K-Y. Prevalence and Related Factors of Depression among the Elderly in Indonesia. International Journal of Gerontology. 2019;13(3):202-6.

4. Mahwati Y. The Relationship between Spirituality and Depression Among the Elderly in Indonesia. Makara Journal of Health Research. 2017;21(1).

5. Mardiana L, Astuti PAS, Suariyani NLP, Wirawan DN. Disability and Lower Social Gradient Increased Risk of Depression Among Pre-Elderly in Indonesia: Finding From a Nationwide Health and Socio-Economic Survey. Asia Pacific Journal of Public Health. 2022;34(5):501-9.

6. Mubasyiroh R, Suryaputri IY, Idaiani S, et al. Mental Health Disorders of the Indonesian People in the Early Stages of the COVID-19 Pandemic: Who is Vulnerable to Experiencing it? International Journal of Mental Health Promotion. 2022;24(5).

7. Abdul Manaf MR, Mustafa M, Abdul Rahman MR, Yusof KH, Abd Aziz NA. Factors Influencing the Prevalence of Mental Health Problems among Malay Elderly Residing in a Rural Community: A Cross-Sectional Study. PLOS ONE. 2016;11(6):e0156937.

8. Ahmad NA, Abd Razak MA, Kassim MS, et al. Association between functional limitations and depression among community-dwelling older adults in Malaysia. Geriatrics & Gerontology International. 2020;20(S2):21-5.

9. Cheah YK, Azahadi M, Phang SN, Abd Manaf NH. Association of Suicidal Ideation with Demographic, Lifestyle and Health Factors in Malaysians. East Asian Arch Psychiatry. 2018;28(3):85-94.

10. Cheah Y, Azahadi M, Phang S, Abd Manaf N. SOCIODEMOGRAPHIC, LIFESTYLE AND HEALTH FACTORS ASSOCIATED WITH DEPRESSION AMONG ADULTS IN MALAYSIA: AN ETHNIC COMPARISON. Journal of Health and Translational Medicine. 2019;22(1).

11. Foong HF, Hamid TA, Ibrahim R, Haron SA. The intersectional effects of ethnicity/race and poverty on health among community-dwelling older adults within multi-ethnic Asian populace: a population-based study. BMC Geriatrics. 2021;21(1):516.

12. Imran A, Azidah AK, Asrenee AR, Rosediani M. Prevalence of depression and its associated factors among elderly patients in outpatient clinic of Universiti Sains Malaysia Hospital. Med J Malaysia. 2009;64(2):134-9.

13. Maideen SFK, Sidik SM, Rampal L, Mukhtar F. Prevalence, Associated Factors and Predictors of Depression among Adults in the Community of Selangor, Malaysia. PLOS ONE. 2014;9(4):e95395.

14. Manaf M, Qureshi A, Loftizadeh M, Ganaegeran K, Yadav H, Al-Dubai S. Factors associated with anxiety and depression among outpatients in Malaysia: a cross-sectional study. Malaysian Journal of Public Health Medicine. 2016;16(3):181-7.

15. Md. Aris MA, Halim NA, Musa R. Prevalence of Depression and Its Associated Risk Factors in the Primary Care Setting in Kuantan. Journal of Advances in Medicine and Medical Research. 2014;4(24):4201-9.

16. Mesbah SF, Sulaiman N, Mohd Shariff Z, Ibrahim Z. Does Food Insecurity Contribute towards Depression? A Cross-Sectional Study among the Urban Elderly in Malaysia. International Journal of Environmental Research and Public Health [Internet]. 2020; 17(9).

17. Rashid A, Tahir I. The Prevalence and Predictors of Severe Depression Among the Elderly in Malaysia. Journal of Cross-Cultural Gerontology. 2015;30(1):69-85.

18. Razali S, Tukhvatullina D, Hashim NA, et al. Sociodemographic Factors of Depression During the COVID-19 Pandemic in Malaysia: the COVID-19 Mental Health International Study. East Asian Arch Psychiatry. 2022;32(4):82-8.

19. Said MA, Thangiah G, Abdul Majid H, et al. Income Disparity and Mental Wellbeing among Adults in Semi-Urban and Rural Areas in Malaysia: The Mediating Role of Social Capital. International Journal of Environmental Research and Public Health [Internet]. 2022; 19(11).

20. Sherina MS, Rampal L, Mustaqim A. The prevalence of depression among the elderly in Sepang, Selangor. Med J Malaysia. 2004;59(1):45-9.

21. Sidik S, Zulkefli N, Shah SA. Factors associated with depression among elderly patients in a primary health care clinic in Malaysia. Asia Pacific Family Medicine. 2003;2(3):148-52.

22. Sidik SM, Rampal L, Afifi M. Physical and mental health problems of the elderly in a rural community of sepang, selangor. Malays J Med Sci. 2004;11(1):52-9.

23. Sidik SM, Arroll B, Goodyear-Smith F, Ahmad R. Prevalence of depression among women attending a primary urban care clinic in Malaysia. Singapore Med J. 2012;53(7):468-73.

24. Sok Yee W, Pei Lin L. Anxiety and Depressive Symptoms among Communities in the East Coast of Peninsular Malaysia: A Rural Exploration. Malaysian Journal of Psychiatry. 2011;20(1).

25. Ting NK, Leong TK. Prevalence and Factors Associated with Depression among Rural Communities in Negeri Sembilan, Peninsular Malaysia. Journal of Scientific Research and Reports. 2014;3(12):1689-702.

26. Vanoh D, Shahar S, Yahya HM, Hamid TA. Prevalence and Determinants of Depressive Disorders among Community-dwelling Older Adults: Findings from the Towards Useful Aging Study. International Journal of Gerontology. 2016;10(2):81-5.

27. Yeoh SH, Tam CL, Wong CP, Bonn G. Examining Depressive Symptoms and Their Predictors in Malaysia: Stress, Locus of Control, and Occupation. Frontiers in Psychology. 2017;8.

28. Cho SM, Saw YM, Saw TN, et al. Prevalence and risk factors of anxiety and depression among the community-dwelling elderly in Nay Pyi Taw Union Territory, Myanmar. Scientific Reports. 2021;11(1):9763.

29. Sasaki Y, Shobugawa Y, Nozaki I, et al. Association between depressive symptoms and objective/subjective socioeconomic status among older adults of two regions in Myanmar. PLOS ONE. 2021;16(1):e0245489.

30. Yamada H, Yoshikawa K, Matsushima M. Geriatric Depressive Symptoms in Myanmar: Incidence and Associated Factors. Journal of Applied Gerontology. 2019;39(11):1230-9.

31. Cheung YB, Ashorn P. Linear growth in early life is associated with suicidal ideation in 18-year-old Filipinos. Paediatric and Perinatal Epidemiology. 2009;23(5):463-71.

32. Anantapong K, Pitanupong J, Werachattawan N. Prevalence of depression, and its associated factors among the elderly in Songkhla Province, Thailand: Two-stage cluster sampling study. Journal of Clinical Gerontology and Geriatrics. 2017;8(2):58-63.

33. Aung MN, Moolphate S, Aung TN, Katonyoo C, Khamchai S, Wannakrairot P. The social network index and its relation to later-life depression among the elderly aged ≥80 years in Northern Thailand. Clin Interv Aging. 2016;11:1067-74.

34. Charoensakulchai S, Usawachoke S, Kongbangpor W, et al. Prevalence and associated factors influencing depression in older adults living in rural Thailand: A cross-sectional study. Geriatrics & Gerontology International. 2019;19(12):1248-53.

35. Haseen F, Prasartkul P. Predictors of depression among older people living in rural areas of Thailand. Bangladesh Medical Research Council Bulletin. 2011;37(2):51-6.

36. Naviganuntana Y, Kerdcharoen N, Rawdaree P. Factors Associated with Depressive Symptoms in Elderly Individuals Living in Urban Communities. Psychol Res Behav Manag. 2022;15:855-64.

37. Peltzer K, Pengpid S. Suicidal ideation and associated factors among clients of primary care and religious care centers in Thailand. Asian Journal of Social Health and Behavior. 2022;5(2):57-62.

38. Prueksaritanond S, Kongsakol R. Biopsychosocial impacts on the elderly from a tsunami-affected community in southern Thailand. J Med Assoc Thai. 2007;90(8):1501-5.

39. Wichaidit W, Prommanee C, Choocham S, Chotipanvithayakul R, Assanangkornchai S. Modification of the association between experience of economic distress during the COVID-19 pandemic and behavioral health outcomes by availability of emergency cash reserves: findings from a nationally-representative survey in Thailand. PeerJ. 2022;10:e13307.

40. Collier KM, Weiss B, Pollack A, Lam T. Explanatory variables for women’s increased risk for mental health problems in Vietnam. Social Psychiatry and Psychiatric Epidemiology. 2020;55(3):359-69.

41. Dao ATM, Nguyen VT, Nguyen HV, Nguyen LTK. Factors Associated with Depression among the Elderly Living in Urban Vietnam. BioMed Research International. 2018;2018:2370284.

42. Do TTH, Nguyen DTM, Nguyen LT. Depressive Symptoms and Their Correlates Among Older People in Rural Viet Nam: A Study Highlighting the Role of Family Factors. Health Services Insights. 2022;15.

43. Duong K, Bao T, Nguyen P, et al. Psychological Impacts of COVID-19 During the First Nationwide Lockdown in Vietnam: Web-Based, Cross-Sectional Survey Study. JMIR Form Res. 2020;4(12):e24776.

44. Giang LT, Nguyen TT, Tran NTT. Factors Associated with Depression among Older People in Vietnam. Journal of Population and Social Studies [JPSS]. 2019;27(2):181 - 94.

45. Hoang VTH, Nguyen HTH. Factors associated with depression, anxiety, and stress symptoms among men in a rural area in Vietnam during COVID-19. Frontiers in Psychiatry. 2022;13.

46. Leggett A, Zarit SH, Nguyen NH, Hoang CN, Nguyen HT. The influence of social factors and health on depressive symptoms and worry: A study of older Vietnamese adults. Aging & Mental Health. 2012;16(6):780-6.

47. Sharma S, Singhal S, Tarp F. Corruption and mental health: Evidence from Vietnam. Journal of Economic Behavior & Organization. 2021;185:125-37.

48. Tran KV, Esterman A, Saito Y, et al. Factors Associated With High Rates of Depressive Symptomatology in Older People in Vietnam. The American Journal of Geriatric Psychiatry. 2022;30(8):892-902.

49. Van NHN, Huyen NTK, Luong NT, Duc DM, Thanh PQ. Factors associated with depression among the elderly living in rural Vietnam 2019: Recommendations to remove barriers of psychological service accessibility. International Journal of Mental Health. 2021;50(2):136-50.

50. Vu HT, Lin V, Pham T, et al. Determining Risk for Depression among Older People Residing in Vietnamese Rural Settings. International Journal of Environmental Research and Public Health [Internet]. 2019; 16(15).

51. Yen Phi HN, Quoc Tho T, Xuan Manh B, et al. Prevalence of depressive disorders in a primary care setting in Ho Chi Minh City, Vietnam: A cross-sectional epidemiological study. The International Journal of Psychiatry in Medicine. 2022;58(2):86-101.

52. Fakhrunnisak D, Patria B. The positive effects of parents’ education level on children’s mental health in Indonesia: a result of longitudinal survey. BMC Public Health. 2022;22(1):949.

53. Isaura ER, Chen Y-C, Adi AC, Fan H-Y, Li C-Y, Yang S-H. Association between Depressive Symptoms and Food Insecurity among Indonesian Adults: Results from the 2007–2014 Indonesia Family Life Survey. Nutrients [Internet]. 2019; 11(12).

54. Patria B. The longitudinal effects of education on depression: Finding from the Indonesian national survey. Frontiers in Public Health. 2022;10.

55. Kim AW, Adam EK, Bechayda SA, Kuzawa CW. Early life stress and HPA axis function independently predict adult depressive symptoms in metropolitan Cebu, Philippines. American Journal of Physical Anthropology. 2020;173(3):448-62.

56. Jittawisuthikul O, Jirapramukpitak T, Sumpowthong K. Disability and late-life depression: a prospective population-based study. J Med Assoc Thai. 2011;94 Suppl 7:S145-52.

57. Peltzer K, Pengpid S. Socioeconomic position and physical and mental health among middle-aged and older adults: Cross-sectional and longitudinal results from a national community sample in Thailand. Journal of Human Behavior in the Social Environment. 2022:1-15.

58. Lueboonthavatchai P. Role of stress areas, stress severity, and stressful life events on the onset of depressive disorder: a case-control study. J Med Assoc Thai. 2009;92(9):1240-9.

59. Mumang AA, Liaury K, Syamsuddin S, et al. Socio-economic-demographic determinants of depression in Indonesia: A hospital-based study. PLOS ONE. 2020;15(12):e0244108.
